# Thermosensitive and antioxidant wound dressings capable of adaptively regulating TGFβ pathways promote diabetic wound healing

**DOI:** 10.1038/s41536-023-00313-3

**Published:** 2023-07-08

**Authors:** Hong Niu, Ya Guan, Ting Zhong, Liang Ma, Mohamed Zayed, Jianjun Guan

**Affiliations:** 1 Department of Mechanical Engineering and Materials Science, Washington University in St. Louis, St. Louis, MO USA; 2 Institute of Materials Science and Engineering, Washington University in St. Louis, St. Louis, MO USA; 3 Department of Internal Medicine, Washington University School of Medicine, St. Louis, MO USA; 4 Department of Surgery, Section of Vascular Surgery, Washington University School of Medicine, St. Louis, MO USA; 5 Department of Radiology, Washington University School of Medicine, St. Louis, MO USA; 6 Department of Biomedical Engineering, Washington University in St. Louis, St. Louis, MO USA

**Keywords:** Regenerative medicine, Drug delivery

## Abstract

Various therapies have been utilized for treating diabetic wounds, yet current regiments do not simultaneously address the key intrinsic causes of slow wound healing, i.e., abnormal skin cell functions (particularly migration), delayed angiogenesis, and chronic inflammation. To address this clinical gap, we develop a wound dressing that contains a peptide-based TGFβ receptor II inhibitor (PTβR2I), and a thermosensitive and reactive oxygen species (ROS)-scavenging hydrogel. The wound dressing can quickly solidify on the diabetic wounds following administration. The released PTβR2I inhibits the TGFβ1/p38 pathway, leading to improved cell migration and angiogenesis, and decreased inflammation. Meanwhile, the PTβR2I does not interfere with the TGFβ1/Smad2/3 pathway that is required to regulate myofibroblasts, a critical cell type for wound healing. The hydrogel’s ability to scavenge ROS in diabetic wounds further decreases inflammation. Single-dose application of the wound dressing significantly accelerates wound healing with complete wound closure after 14 days. Overall, using wound dressings capable of adaptively modulating TGFβ pathways provides a new strategy for diabetic wound treatment.

## Introduction

Diabetes affects more than 34 million people in the United States alone^1^. Elderly individuals represent a large fraction of this population, with approximately 25% of people over the age of 65 reported to have diabetes. This is currently the leading cause of non-traumatic lower limb amputation, predominantly due to the development of chronic foot wounds that are prone to infection and severe tissue damage^1,2^. These wounds are non-healing wounds and thus are challenging to manage in clinics. Diabetic wounds deviate from the typical physiological process of normal wound healing. They become trapped in the inflammatory phase, resulting in prolonged inflammation. Moreover, diabetic wounds display significant dysfunction in angiogenesis and remodeling processes, as well as impaired functionality of skin cells. These pathophysiological alterations significantly contribute to the chronic nature of diabetic wounds^1,2^. Various therapies have been explored to treat diabetic wounds, such as hyperbaric oxygen treatment^3–6^, cell therapy^7–12^, growth factor delivery^13–26^, and various types of wound dressing^27–43^. However, current therapies are not efficient in resolving chronic inflammation, delayed angiogenesis, and skin cell dysfunctions (particularly migration), which are key intrinsic barriers to diabetic wound healing^44–49^. In addition, cell therapy faces challenges such as off-the-shelf availability of cells, immune response, regulatory compliance, and ethical considerations. Growth factor delivery has limitations such as short half-life, high doses, and difficulty in delivering multiple growth factors to sequentially regulate each stage of wound healing.

To address these causes, control of TGFβ signaling is crucial because it plays direct and differential roles in delaying or promoting diabetic wound healing^50–52^. The TGFβ1/p38 pathway is directly associated with prolonged tissue inflammation and impaired skin cell migration^53–58^. The reduced endothelial cell migration leads to delayed angiogenesis^53–58^. De-activation of the TGFβ1/p38 pathway has been found to reduce skin inflammation and promote epithelialization and granulation tissue formation^53–58^. Meanwhile, TGFβ1/Smad2/3 pathway is required to regulate myofibroblasts, which are critical cells for wound healing^50–52^. Thus, inhibition of the TGFβ1/p38 pathway without impacting the TGFβ1/Smad2/3 pathway would presumably accelerate diabetic wound healing. However, current approaches have not been able to achieve this goal^44–48^, and new treatments are critically needed.

To reduce the deleterious impact of TGFβ1 on wound healing or tissue repair, systemic delivery of TGFβ inhibitors or anti-TGFβ antibodies have been commonly used to decrease the amount of active TGFβ1^59–61^. While these approaches have shown some therapeutic benefits, relatively low efficacy and bioavailability have hindered their widespread clinical adoption^50–52^. The low efficacy may reflect the fact that these inhibitors and antibodies only decrease the amount of active TGFβ1, but cannot fundamentally inhibit the TGFβ1/p38 pathway. On the other hand, TGFβ receptor (TβR) inhibitors have the potential to fundamentally inhibit the TGFβ1/p38 pathway by blocking TGFβ1 from binding to TβRs on cells. While various TβR inhibitors exist, most are small-molecule inhibitors used for cancer therapy^62,63^. One potential challenge in using these inhibitors for diabetic wound healing is their effective dosages can be toxic to skin cells^62,63^. They can not only bind to the cell surface receptors, but also interact with intracellular proteins, thereby increasing the risk of cytotoxicity^64^. Indeed, cutaneous toxicity has been reported for approximately 90% of the FDA-approved small-molecule inhibitors used in clinics^65^. In addition, existing TβR inhibitors have not shown the capability of inhibiting the TGFβ1/p38 pathway while not inferring with the essential TGFβ1/Smad2/3 pathway for diabetic wound healing. Despite these limitations, collective preclinical studies and clinical trials suggest that targeting TGFβ signaling remains an important therapeutic strategy in accelerating diabetic wound healing^50–52,66,67^.

In our study, we developed a peptide-based TβR2 inhibitor (PTβR2I) to block the TGFβ signaling pathway. Interestingly, we found that PTβR2I exhibits the capability of adaptively modulating the TGFβ/p38 pathway under hyperglycemic conditions, without impacting the TGFβ1/Smad2/3 pathway. To further advance the clinical application of our findings, we incorporated the PTβR2I peptide into a novel wound dressing. Applied topically or by injection, the wound dressing quickly solidifies to retain the drug on the wound bed, where it gradually releases PTβR2I to continuously block the TGFβ1/p38 pathway and enhance skin cell migration, stimulate angiogenesis, and attenuate tissue inflammation. The hydrogel can scavenge ROS to reduce wound inflammation. Here, we demonstrate that PTβR2I inhibits the TGFβ1/p38 pathway under high glucose conditions without substantially impacting the TGFβ1/Smad2/3 pathway, which is responsible for the formation of myofibroblasts^50,52^.

## Results

### Binding affinity of PTβR2I and its effect on skin cells

**Fig. 1:**
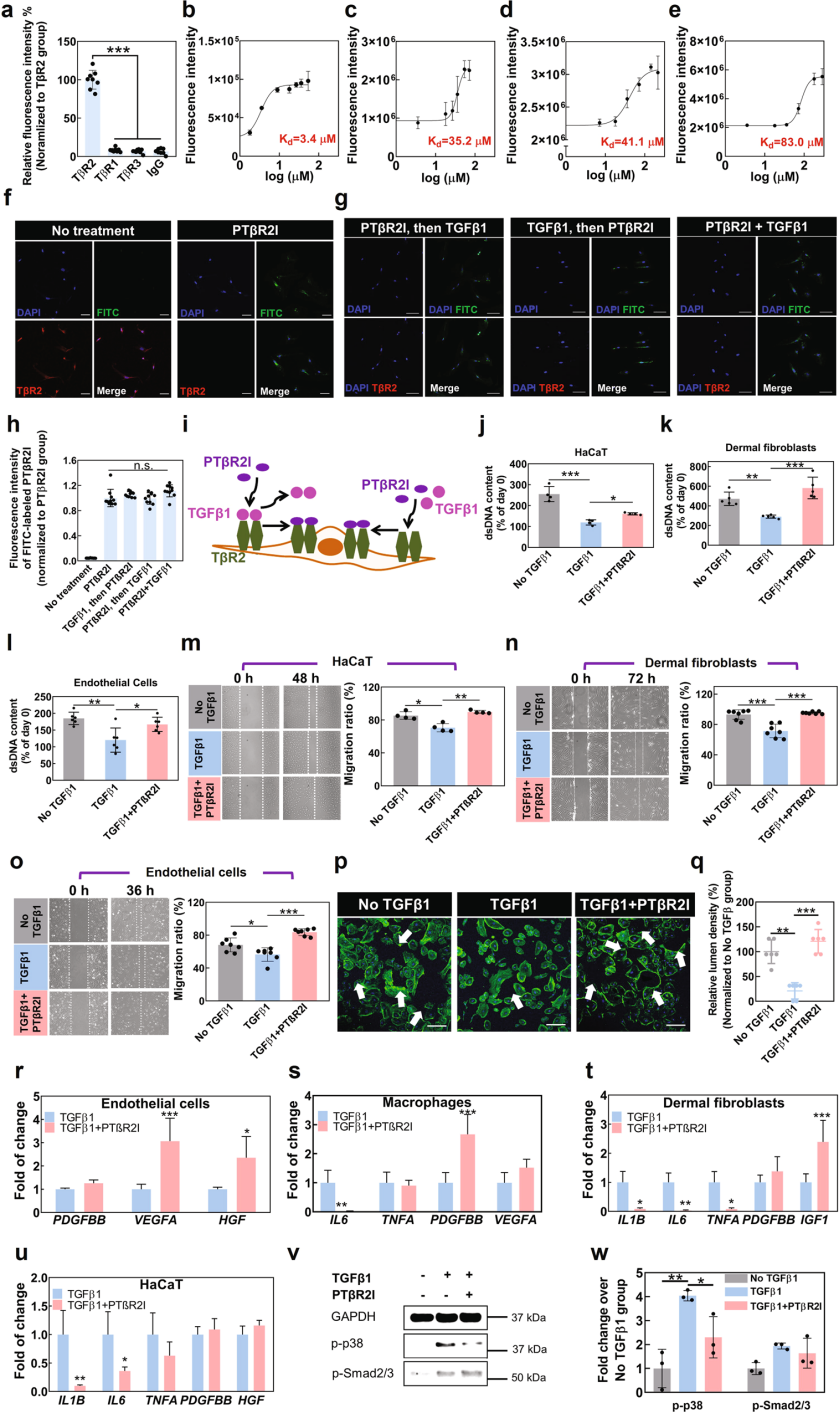
Multifunction of TβR2 binding peptide PTβR2I in different cells. **a** Binding affinity between PTβR2I and TβR1/TβR2/TβR3/IgG, tested by the binding assay (*n* = 8). **b**–**e** K_d_ values of PTβR2I in binding with **b** TβR2, **c** TβR1, **d** TβR3, and **e** IgG measured by an ELISA-like binding assay (*n* = 8). **f** PTβR2I binding specificity was tested by staining the HDFs with an anti-TβR2 antibody. The PTβR2I treated group showed evident expression of FITC, and no unbound TβR2 was observed. Scale bar =50 µm. **g** The competitive binding affinity to TβR2 was also examined with a total treatment time of 48 h. Scale bar = 50 µm. **h** The competitive binding between PTβR2I and TGFβ1 was determined using fluorescence intensity measurements by a plate reader (excitation/emission = 485/535 nm). The non-PTβR2I -treated group served as a control. The total time for all treatments was 48 h (*n* = 10). *p* > 0.05 for any pair of the groups with the addition of PTβR2I. **i** Schematic showing how PTβR2I binds to TβR2 on HDF and how it surpasses TGFβI in binding ability. **j** dsDNA of HaCaT treated with TGFβ1 and PTβR2I at day 3 (*n* = 4). **k** dsDNA of dermal fibroblasts at day 3 (*n* = 5). **l** dsDNA of endothelial cells at day 3 (*n* = 6). **m** Representative images and quantification of migration using a scratch assay for HaCaT at 0 and 48 h (*n* = 4). **n** Representative images and quantification of migration for dermal fibroblasts at 0 and 72 h (*n* = 7). **o** Representative images and quantification of migration for endothelial cells at 0 and 36 h (*n* = 7). **p** Representative images of endothelial cell lumen formation (lumens are indicated by white arrows) 24 h post-treatment. Cytoskeleton was stained by F-actin. Scale bar = 50 µm. **q** Quantification of lumen density based on the images (*n* = 6). **r** Folds of change for *PDGFBB*, *VEGFA*, and *HGF* in endothelial cells. **s** Folds of change for *IL6*, *TNFA*, *PDGFBB*, and *VEGFA* expressed from macrophages. **t** Folds of change for *IL1B*, *IL6*, *TNFA*, *PDGFBB*, and *IGF1* in dermal fibroblasts. **u** Folds of change for *IL1B*, *IL6*, *TNFA*, *PDGFBB*, and *HGF* in HaCaT treated with no TGFβ1, TGFβ1, or PTβR2I and TGFβ1. **v**, **w** Immunoblotting of p-p38 and p-Smad2/3 derived from dermal fibroblasts. GAPDH was used as a loading control (*n* = 3). The cells were treated with no TGFβ1, TGFβ1, and PTβR2I with TGFβ1 under high glucose. All data demonstrated as mean ± standard deviation. Data were analyzed by one-way ANOVA with Bonferroni post-test (n.s. *p* > 0.05, *p < 0.05, **p < 0.01, ***p < 0.001).

We synthesized a peptide ECGLLPVGRPDRVWRLCK-FITC (PTβR2I), based on the sequence from a phage display library that binds specifically to TβR2, and the interactions between TβR2 and TGFβ1^68–70^. This peptide contains amino acids in the TGFβ1 C-terminal domain that are critical for high binding affinity to TβR2, V (residue 92), R (residue 94), and V (residue 98). In addition, the 2 cysteine residues act to stabilize the peptide structure. We first examined the specific binding of PTβR2I with TβR2 using an ELISA-like binding assay. TGFβ receptor I (TβR1), TGFβ receptor III (TβR3), and immunoglobulin G (IgG) were used as controls. We confirmed that PTβR2I has a remarkably higher binding affinity to TβR2 than its binding affinities to TβR1, TβR3, or IgG (*p* < 0.001, Fig. 1a). The dissociation constant (K_d_) of the PTβR2I binding to TβR2 is 3.4 µM, more than 10 times lower than those for PTβR2I binding to TβR1, TβR3, and IgG (Fig. 1b–e). This finding was further validated on the cellular level. We used human dermal fibroblasts (HDFs) to study the binding affinity of PTβR2I to TβR2 under a glucose level of 4.5 g L^−1^, which mimics the hyperglycemic conditions of diabetes. With PTβR2I added, TβR2 binding sites were occupied, so the TβR2 could not be detected by immunofluorescence (Fig. 1f), and instead, the signal from PTβR2I that is tagged with FITC was clearly observed on the cells.

We also performed a competitive binding test between PTβR2I and TGFβ1 at the cellular level. We found that the fluorescence of TβR2 was not detected with PTβR2I treatments, either pre-, post-, or simultaneous to TGFβ1 treatments (Fig. 1g). The fluorescent intensity of FITC-labeled PTβR2I was consistent with whether PTβR2I or TGFβ1 was first bound to TβR2 (Fig. 1h and Supplementary Fig. 1). These results demonstrated that PTβR2I has a higher binding affinity to TβR2 than TGFβ1. Once PTβR2I binds to TβR2, TGFβ1 cannot bind to the receptor. More interestingly, PTβR2I is able to pull off the bound TGFβ1 from the receptors and then competitively occupy the binding sites instead (Fig. 1i). In addition, we tested the cytotoxicity of PTβR2I in different concentrations on several major types of cells, including HDF, human keratinocytes HaCaT cells, and human arterial endothelial cells (HAEC) (Supplementary Fig. 2a–c). With PTβR2I treatment, we observed no decrease in cell viability at the test concentrations (1 to 100 μg mL^−1^), indicating high cytocompatibility and biotolerance to PTβR2I.

During diabetic wound healing, keratinocytes, fibroblasts, and endothelial cells are respectively involved in re-epithelialization, dermal formation, and angiogenesis, yet their proliferation and migration are compromised in the diseased environment due to the high glucose concentration and upregulated TGFβ1^50,71^. We investigated whether PTβR2I can restore the proliferation and migration of keratinocytes, fibroblasts, and endothelial cells under these conditions. We found significantly improved proliferation of HaCaTs, HDFs, and HAECs in the groups with PTβR2I (Fig. 1j–l, *p* < 0.05 for HaCaTs, *p* < 0.001 for HDFs, and *p* < 0.05 for HAECs). Specifically, for the HaCaT cells, the relative dsDNA content was increased from 119.5% in the TGFβ1 group to 160.7% in the TGFβ1 + PTβR2I group. For the HAECs, the PTβR2I treatment increased the relative dsDNA content from 120.3% to 167.1%. The most significant proliferation was found for the HDFs where the relative dsDNA content was increased over 2-fold after PTβR2I treatment.

To determine how PTβR2I influences the migration of HDFs, HaCaTs, and HAECs under high glucose and TGFβ1 conditions, a 2D scratch assay was used^72,73^. TGFβ1 significantly decreased the migration of all three cell types (Fig. 1m–o). After the cells were treated with PTβR2I, a significantly higher number of cells migrated. These results demonstrate that PTβR2I treatment can significantly increase the proliferation and migration of keratinocytes, fibroblasts, and endothelial cells.

### Effect of PTβR2I on skin cell morphogenesis and secretome

In diabetic wounds, angiogenesis is delayed due to impaired endothelial morphogenesis^74,75^, and impeded expression of angiogenic growth factors such as vascular endothelial growth factor (VEGF)^17^, platelet-derived growth factor-BB (PDGFBB)^76^, and hepatocyte growth factor (HGF)^77^. We first examined the effect of PTβR2I treatment on endothelial morphogenesis by performing an in vitro lumen formation assay using HAECs. The lumen density in the TGFβ1 group was much lower than that in the group without TGFβ1 (*p* < 0.001), whereas PTβR2I reversed the situation and significantly increased the lumen density (Fig. 1p, q). We further investigated whether PTβR2I treatment affected the expression of angiogenic growth factors from HaCaTs, HDFs, HAECs, and THP-1-derived CD86^+^ M1 macrophages. We found that the gene expressions of *VEGFA* and *HGF* in PTβR2I-treated HAECs (Fig. 1r), and *PDGFBB* in PTβR2I-treated M1 macrophages (Fig. 1s) were significantly enhanced. Notably, PTβR2I treatment also significantly upregulated the expression of prosurvival insulin like growth factor 1 (*IGF1*) in HDFs (Fig. 1t).

Diabetes is characterized by chronic inflammation, evidenced by the increased expression of proinflammatory cytokines such as interleukin-6 (IL6)^78^, interleukin-1β (IL1β)^79,80^, and tumor necrosis factor-α (TNFα)^81^. Thus, we evaluated whether PTβR2I modulated the expressions of inflammatory cytokines in HaCaT, HDFs, HAECs, and THP-1-derived CD86^+^ M1 macrophages. These cells are major sources of proinflammatory cytokines during diabetic wound healing^78^. At the mRNA level, after PTβR2I treatment, the expressions of *IL6* in M1 macrophages, *IL1B, IL6*, and *TNFA* in HDFs, and *IL1B* and *IL6* in HaCaTs were all significantly reduced (Fig. 1s–u).

### Mechanisms of action of PTβR2I under high glucose condition

Collectively, our data demonstrate that PTβR2I treatment promoted the proliferation and migration of keratinocytes, dermal fibroblasts, and endothelial cells, stimulated endothelial morphogenesis, increased angiogenic growth factor expression in endothelial cells and macrophages, and reduced the expression of proinflammatory cytokines in dermal fibroblasts (*IL1B*, *IL6*, and *TNFA*), keratinocytes (*IL1B* and *IL6*), and macrophages (*IL6*). These actions are impaired by upregulated TGFβ1 and the high glucose condition in diabetic wounds. To delineate the underlying mechanism, we conducted immunoblotting study on HDFs under high glucose and TGFβ1 conditions (Fig. 1v, w). We targeted the TGFβ1/p38 pathway because it is directly associated with impaired cell migration in diabetic wounds^53–58^. In addition, previous studies have shown that inhibition of TGFβ1/p38 signaling decreased inflammation during diabetic wound healing^53–57^. We observed that, relative to no TGFβ1 group, TGFβ1-treated cells displayed a near 4-fold increase in the phosphorylation of p38 (p-p38), whereas PTβR2I with TGFβ1 significantly downregulated p-p38 (*p* < 0.05). To further confirm the role of PTβR2I in de-activating the p38 pathway, we treated the HDFs with a p-38 inhibitor (SB202190), which notably downregulated the p-p38 expression (Supplementary Fig. 3).

Myofibroblasts are critical for wound healing, and the TGFβ1/Smad2/3 pathway represents a primary signaling pathway to drive the myofibroblast formation from fibroblasts. We investigated whether PTβR2I treatment affected the TGFβ1/Smad2/3 pathway and myofibroblast formation under high glucose conditions. Interestingly, the PTβR2I treatment did not substantially influence p-Smad2/3 expression (Fig. 1v). This is consistent with α-SMA expression where the ratio of α-SMA^+^ cells did not change with the addition of PTβR2I (Supplementary Fig. 4).

### Antioxidant wound dressings capable of releasing PTβR2I

**Fig. 2:**
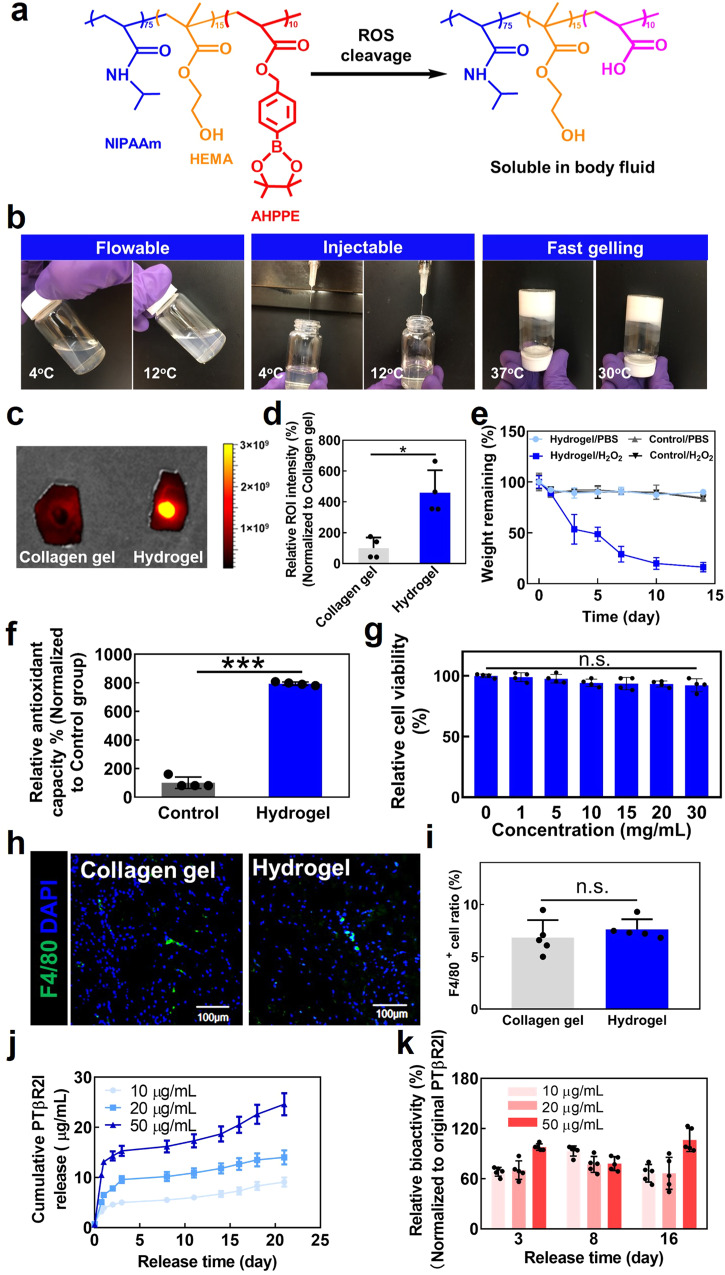
Characterization of ROS-responsive hydrogel as a vehicle for loading PTβR2I peptide. **a** Chemical structure of polymer (NIPAAm-*co*-HEMA-*co*-AHPPE) and its accelerated degradation with ROS. The final degraded product is soluble in body fluid. **b** The hydrogel solution is flowable and injectable at both 4 °C and 12 °C, and quickly forms a solid gel at 30 °C and 37 °C. **c** Retention of PTβR2I encapsulated in the developed hydrogel in the wound area after 24 h, observed using IVIS, with comparative collagen gel retention results. **d** Quantification of drug retention by relative ROI intensity derived from IVIS images (*n* = 3). **e** Degradation of the hydrogel in PBS with or without 1 mM H_2_O_2_ for 14 days. A non ROS-responsive hydrogel was used as a control. **f** In vitro total antioxidant capacity of the hydrogel. **g** Cytotoxicity of different concentrations of the degraded product to dermal fibroblasts, was evaluated by MTT assay (*n* = 4). **h** In vivo biocompatibility of the ROS-sensitive hydrogels, examined by F4/80 staining (green) on tissue samples with subcutaneously injected hydrogel after 7 days. Nuclei were stained with DAPI (blue). **i** Quantification of F4/80^+^ cell ratio based on the images (*n* = 5). **j** In vitro release profiles of 3 different concentrations of PTβR2I in ROS-responsive gel for 21 days (*n* = 4). **k** Bioactivity of PTβR2I released from ROS-responsive gel at days 3, 8, and 14 (*n* = 5). All data are shown as mean ± standard deviation. Data were analyzed by one-way ANOVA with the Bonferroni post-test (n.s. *p* > 0.05, *p < 0.05).

We further developed a wound dressing by encapsulating PTβR2I in an injectable, thermosensitive, and ROS-scavenging hydrogel. The hydrogel was based on N-isopropylacrylamide, 2-hydroxyethyl methacrylate, and 4-(acryloxymethyl)-phenylboronic acid pinacol ester (AHPPE) (Fig. 2a). These three components are respectively responsible for thermosensitivity, increased hydrophilicity, and ROS-scavenging. The ^1^H-NMR spectrum clearly displayed the characteristic peaks for each component (Supplementary Fig. 5), and the calculated ratio of the four components was 76.3/14.2/9.5 (Supplementary Fig. 6). The hydrogel solution (6 wt%) had a thermal transition temperature of 17.8 °C determined by rheological test (Supplementary Fig. 7). The 4 °C (hydrogel solution storage temperature) and 12 °C solutions were flowable and easily injected through a 27 G needle (Fig. 2b). After the hydrogel solution was solidified at 37 °C (typical body temperature), and the formed hydrogel was incubated in 37 °C Dulbecco’s phosphate-buffered saline (DPBS) for 24 h, it exhibited a water content of 73.2 ± 5.1%. The 4 °C solution quickly gelled at 30 °C (typical wound temperature, within 12 s) and 37 °C (within 6 s). Thus, after being applied into wounds, the wound dressing can solidify rapidly to efficiently confine PTβR2I within the tissue. To confirm this function, we applied the wound dressing topically into mouse wounds. Collagen (gelation time ~21 min) encapsulating PTβR2I was used as a control. The wound dressing solidified quickly in the tissue without any leak. In contrast, a substantial amount of collagen/ PTβR2I was leaked out. When examined after 24 h, the wound dressing had remarkably improved PTβR2I retention compared to retention by collagen/PTβR2I (Fig. 2c, d). These results show that the developed wound dressing is suitable for sustainably delivering drugs to wounds.

To evaluate the ROS responsiveness of the hydrogel, we performed a degradation study in DPBS containing hydrogen peroxide (H_2_O_2_) to mimic excessive oxidative stress in diabetic wounds (Fig. 2e). The hydrogel showed a significantly higher weight loss in the DPBS with H_2_O_2_ than in the DPBS without H_2_O_2_ during the 2-week study period. In contrast, the control, non-ROS responsive hydrogel did not exhibit H_2_O_2_-dependent degradation. To further evaluate the ROS scavenging capability of the hydrogel, a total antioxidant capacity assay was conducted. Compared with the non-ROS responsive control hydrogel, the ROS-responsive hydrogel demonstrated an approximately 8-fold increase in the total antioxidant capacity (Fig. 2f).

To assess the biocompatibility of the hydrogel, we synthesized the final product after the phenylboronic acid pinacol ester moiety in the hydrogel was totally removed by ROS (Fig. 2a), and measured the cell viability of HDFs cultured with the final product. The synthesized final product, with a thermal transition temperature of 43 °C, could thus dissolve in body fluid at 37 °C. HDFs cultured with or without the final product retained the same viability, even when the concentration was as high as 30 mg mL^−1^ (Fig. 2g). To further examine the hydrogel’s biocompatibility, we injected the hydrogel subcutaneously into the dorsal side of C57BL/6 J mice. Collagen gel was used as a control group. Seven days after implantation, the tissues injected with either collagen or the hydrogel exhibited similar densities of F4/80^+^ macrophages (Fig. 2h, i). The above results demonstrate that the hydrogel is a biocompatible wound dressing.

PTβR2I was gradually released from the wound dressing during a 21-day release study (Fig. 2j). An initial burst release was observed on the first day, followed by a slower and sustained release until day 21. The release kinetics depended on the amount of PTβR2I loaded into the wound dressings: higher loading dosages resulted in greater amounts of released PTβR2I. To determine the bioactivity of the released PTβR2I, we performed a cell binding assay using HDFs treated with the PTβR2I amounts released on days 3, 8, and 14 for loading dosages of 10, 20, and 50 µg mL^−1^. The PTβR2I released at all three timepoints from all the loading dosages remained bioactive (Fig. 2k).

### Effect of PTβR2I-releasing wound dressings on wound closure

**Fig. 3:**
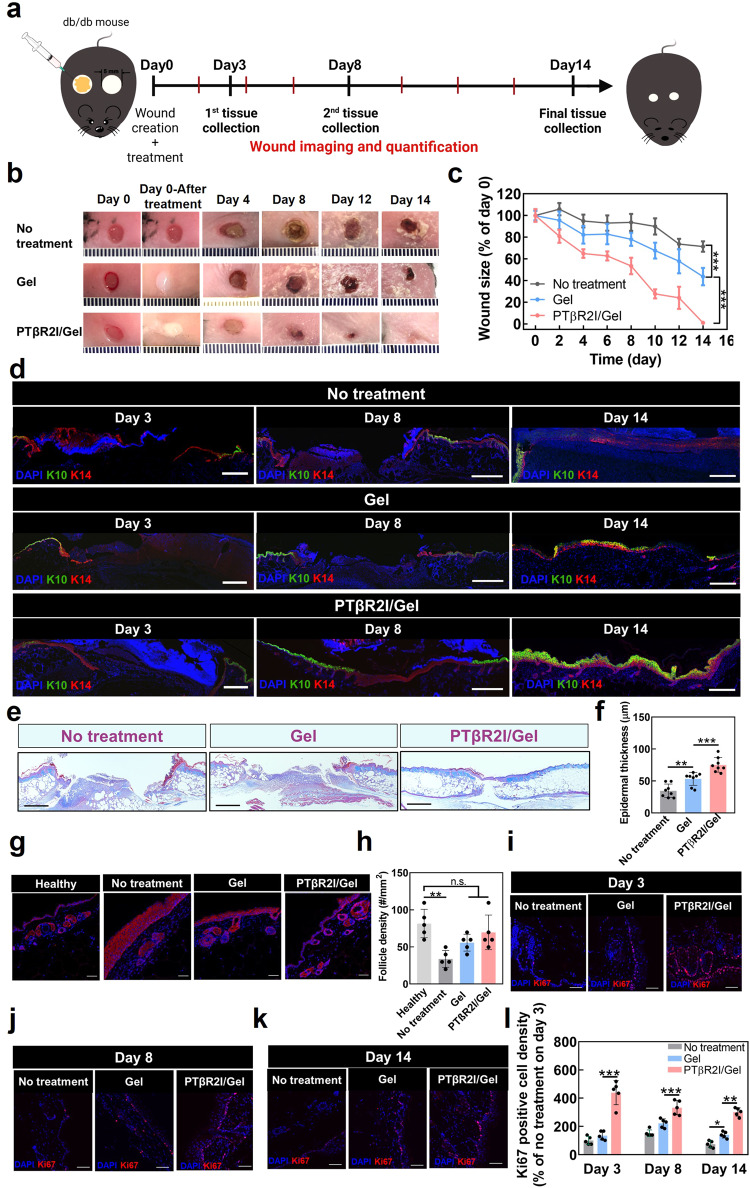
PTβR2I encapsulated in ROS-responsive gel accelerated wound healing in diabetic mice, with improved keratinocyte migration and hair follicle formation in vivo. **a** Timeline of diabetic mouse wound healing. **b** Representative images of wounds taken from day 0 to day 14. Wounds were created using 5 mm biopsy punches on the dorsal skin of *db/db* mice and treated with hydrogel and PTβR2I/Gel topically and subcutaneously. **c** Wound sizes over 14 days course of each treatment. Wound size ratios were normalized to day 0 (*n* ≥ 6). **d** Cytokeratin 10 and 14 staining illustrated enhanced keratinocyte migration at days 3, 8, and 14 in the PTβR2I/Gel group, compared with the No treatment group and gel-only group. Scale bar = 500 µm for days 3 and 8. Scale bar = 200 µm for day 14. **e** Masson’s trichrome stained wound sections on day 8 of no treatment, treatment with gel, and treatment with PTβR2I encapsulated in the gel. Scale bar = 500 µm. **f** Quantification of epidermal thickness at day 8 (*n* = 8). **g** Immunohistochemical (IHC) staining of K14 (red) in the wounded region on day 14. Scale bars = 50 μm. **h** Hair follicle density in the wounded area at day 14 (*n* = 5). **i**–**k** Representative images of proliferated cells in the wound area at days 3, 8, and 14, were made using anti-Ki67 antibody. Scale bar = 50 μm. **l** Quantification of proliferated cell (Ki67^+^) density (*n* = 5). All data are shown as mean ± standard error **c** or mean ± standard deviation **f**, **h**, and **l**. Data were analyzed by one-way ANOVA with Bonferroni post-test (n.s.*p* > 0.05, **p < 0.01, ***p < 0.001).

The efficacy of the PTβR2I-releasing wound dressing in accelerating wound healing was evaluated using *db/db* mice, a strain that represents a type II diabetes model characterized by obesity, hyperglycemia, and deficient wound closure. The wound dressings were administrated onto full-thickness excisional wounds (Fig. 3a). The wounds treated with PTβR2I-releasing wound dressing (PTβR2I/Gel group) exhibited faster wound closure than either those without treatment (No-treatment group), or those treated with wound dressing without PTβR2I (Gel group) (Fig. 3b, c). Starting from day 4, the wound size in the PTβR2I/Gel group was significantly smaller than in the No-treatment and Gel groups (*p* < 0.05). At day 14, the wounds in the PTβR2I/Gel group were fully closed, while those in the No-treatment and Gel groups remained unclosed with wound sizes of 71.4 ± 5.9% and 43.7 ± 8.0%, respectively. Interestingly, treating the wounds with the wound dressings without PTβR2I (Gel group) also significantly decreased the wound size at day 14 (*p* < 0.001), demonstrating that the hydrogel alone could accelerate wound closure.

To determine how the PTβR2I-releasing wound dressings affected the wound reepithelialization process, wounds were harvested at days 3, 8, and 14 and stained for basal keratinocytes (positive for cytokeratin 14 (K14^+^)) and spinous keratinocytes (positive for cytokeratin 10 (K10^+^)). With increasing days of wound healing, K14^+^ basal keratinocytes moved toward spinous layers and differentiated into K10^+^ spinous keratinocytes^82^. By day 3, compared to observations of wounds dressed without PTβR2I (the Gel group) and the No-treatment group, for wounds dressed with PTβR2I (the PTβR2I/Gel group), more K14^+^ basal keratinocytes had migrated to the wound area (Fig. 3d). By day 8, the middle stage of wound healing, the K10^+^ and K14^+^ keratinocytes covered the entire wound in the PTβR2I/Gel group, while the keratinocytes had migrated more slowly in the wounds in the Gel and No-treatment groups, where a large region was without keratinocyte coverage. In addition, the PTβR2I/Gel group formed a more mature spinous layer composed of K10^+^ keratinocytes. Consistent with the keratinocyte migration results, the wounds in the PTβR2I/Gel group exhibited the thickest epidermis on day 8 (Fig. 3e, f). The timepoint was determined to reflect the most significant differences in the proliferative stage of wound healing. On day 14, a clearly stratified epithelium was observed in the PTβR2I/Gel group, indicating that the reepithelialization was fully complete (Fig. 3e). In contrast, the wounds in the Gel and No-treatment groups were not fully covered by keratinocytes. Notably, larger areas of wounds in the Gel group had keratinocyte coverage than in the No-treatment group. Moreover, hair follicle development in the basal layer of the epidermis was promoted along with accelerated reepithelization. On day 14, the wounds dressed with PTβR2I/Gel exhibited the highest density of K14^+^ hair follicles, which is a hallmark of successful healing at the end stage (Fig. 3g, h)^83^, similar to that in surgically unwounded *db/db* mice (*p* > 0.05). These results demonstrate that wound dressings based on the PTβR2I and ROS scavenging hydrogel were able to effectively accelerate keratinocyte migration and hair follicle formation, leading to faster reepithelization in diabetic mice.

The improved wound closure rate might also be the result of enhanced cell proliferation. To elucidate the relationship, we stained the wounds on days 3, 8, and 14 with a cell proliferation marker Ki67. On day 3 (early stage of wound healing), compared to the Gel and No-treatment groups, the PTβR2I/Gel group exhibited a remarkably greater density of Ki67^+^ proliferating cells in both the epidermal and dermal layers (Fig. 3i, l. *p* < 0.001). The density of proliferating cells had decreased in the PTβR2I/Gel group in the middle (day 8) and late (day 14) stages of wound healing, yet it remained significantly higher than in the other two groups (Fig. 3j, k, and i. *p* < 0.001). Notably, cell proliferation was also promoted in the Gel group, where the Ki67^+^ cell density was greater than in the No-treatment group (*p* > 0.05 on days 3 and 8, and *p* < 0.05 on day 14). These results demonstrate that the PTβR2I and the ROS-scavenging hydrogel in the wound dressings can effectively stimulate cell proliferation in the epidermal and dermal layers during diabetic wound healing.

### Effect of PTβR2I-releasing wound dressings on angiogenesis

**Fig. 4:**
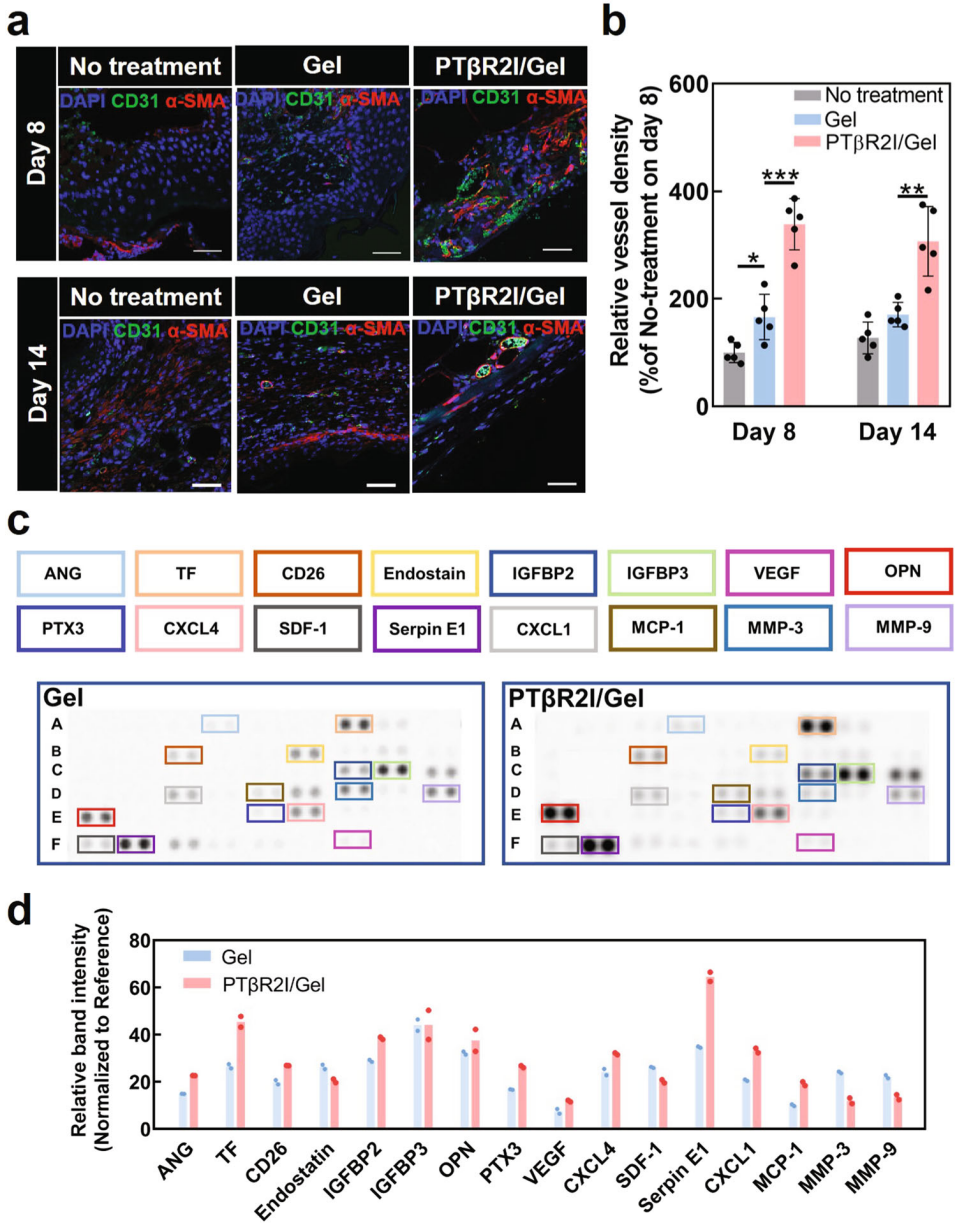
Treatment of PTβR2I-releasing wound dressings stimulated angiogenesis and angiogenic growth factor secretion in diabetic wounds. **a** Representative days 8 and 14 immunofluorescence stain images of vessel formation, made using CD31 and α-SMA (nuclei in blue using DAPI). Scale bar = 50 μm. **b** Relative CD31+ and α-SMA+ vessel density that is normalized to that of the No treatment group at day 8 (*n* = 5). The vessel density was quantified based on the images first followed by normalization. **c** The image of dots obtained from the angiogenesis protein array was performed using the tissue samples collected on day 8 post-treatment. **d** Band intensities of proteins in the angiogenesis array. All data demonstrated as mean ± standard deviation. Data were analyzed by one-way ANOVA with Bonferroni post-test **p < 0.01, ***p < 0.001).

Angiogenesis is critical for healing diabetic wounds. To evaluate whether PTβR2I-releasing wound dressings stimulated angiogenesis, we compared blood vessels in the wounds for the PTβR2I/Gel, Gel, and No-treatment groups at the middle (day 8) and late (day 14) stages of the wound healing (Fig. 4a, b). By day 8, the wounds treated with the Gel group exhibited promoted angiogenesis, with a significantly higher vessel density than the No-treatment group (*p* < 0.05), and a higher vessel density by day 14. These observations demonstrate that the ROS-scavenging hydrogel itself was able to enhance angiogenesis. Treatment with PTβR2I/Gel wound dressing more pronouncedly stimulated angiogenesis, as evidenced by a significantly higher vessel density than in the Gel and No-treatment groups at two stages of the wound healing (*p* < 0.001 at day 8, and *p* < 0.01 at day 14), demonstrating that the released PTβR2I can effectively induce angiogenesis.

To further assess the role of released PTβR2I in promoting tissue angiogenesis, we used a protein array assay to examine the expression of major angiogenic factors in diabetic wounds (Fig. 4c). We found that the PTβR2I/Gel group substantially increased the expression of angiogenic proteins, such as VEGF^84,85^, angiogenin (ANG)^86^, tissue factor (TF)^87^, and serpin E1 (Fig. 4c, d)^88^. These results demonstrate that PTβR2I upregulates angiogenic factor expressions in diabetic wounds.

### Effect of PTβR2I-releasing wound dressings on inflammation

**Fig. 5:**
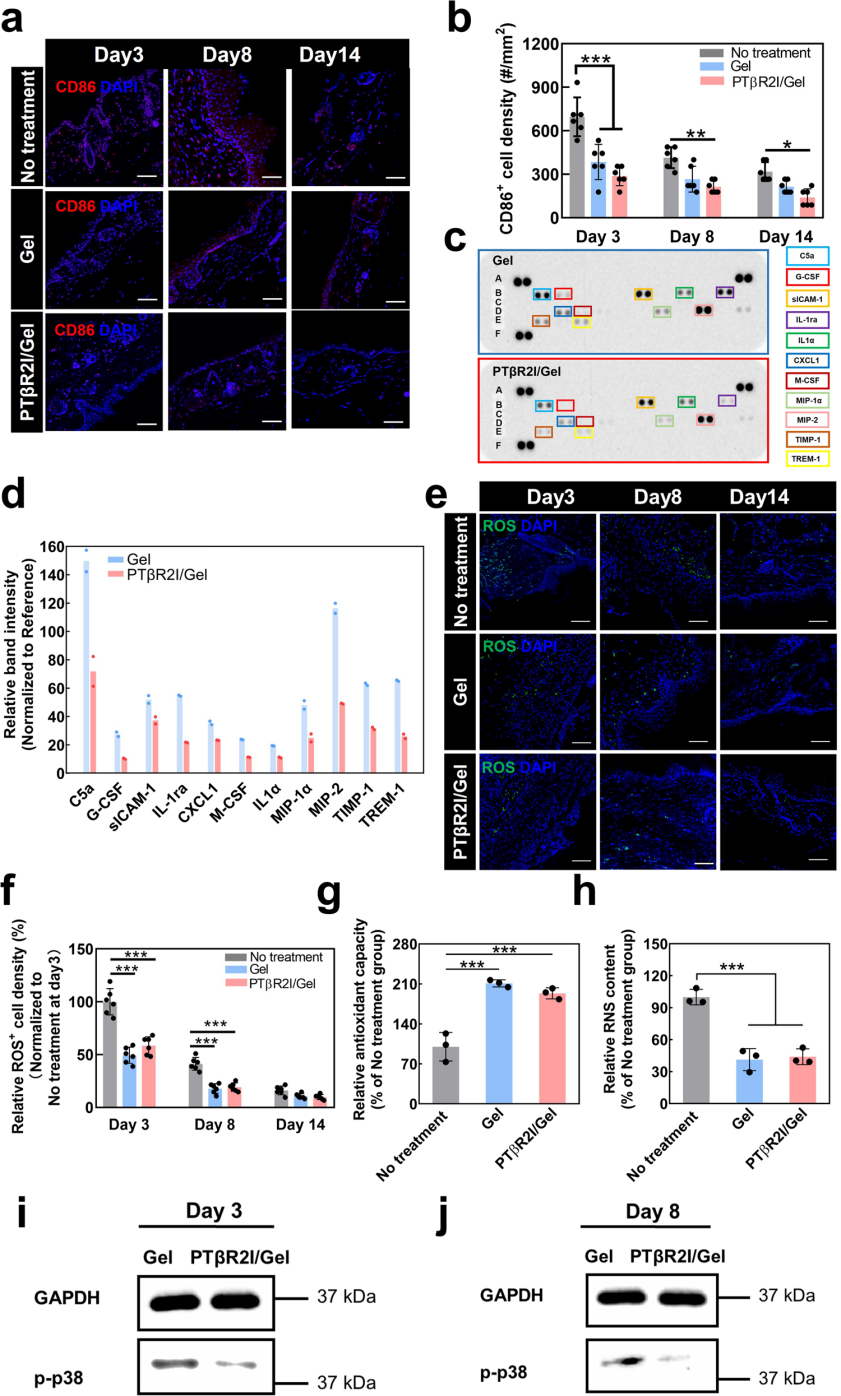
Treatment of PTβR2I-releasing wound dressings alleviated inflammatory response by decreasing M1 macrophage density, the expression of pro-inflammatory cytokines, and ROS content. **a** Representative IHC images, using anti-CD86, of tissue sections at days 3, 8, and 14 post treatment. Nuclei were stained with DAPI. Scale bar = 50 µm. **b** Quantification of CD86^+^ cell density in the wounded area (*n* = 6). **c** Protein array analysis of pro-inflammatory cytokines for tissue samples collected on day 3 post treatment. **d** Quantification summary for the cytokine array. All data are shown as mean ± standard deviation. **e** ROS staining using CellROX deep red of tissue sections harvested 3, 8, and 14 days post treatment. Scale bar = 50 µm. **f** Quantification of relative ROS^+^ cell density (*n* = 6). **g** Quantification of total antioxidant capacity from skin tissues collected from wound sites at day 3 (*n* = 3). **h** RNS content of skin tissues collected from wound sites at day 3 (*n* = 3). **i**, **j** Western blot analysis of p-p38 in the wounded skin of *db/db* mice on days 3 and 8. GAPDH was used as a loading control. All data demonstrated as mean ± standard deviation. Data were analyzed by one-way ANOVA with Bonferroni post-test (*p < 0.05, **p < 0.01, ***p < 0.001).

To determine whether PTβR2I-releasing wound dressings provoked an inflammatory response in diabetic wounds, we quantified the M1 macrophage density and proinflammatory cytokine expression at different stages of wound healing (Fig. 5a, b). Compared to the No-treatment group on days 3, 8, and 14, treatment with the ROS-scavenging hydrogel alone (Gel group) substantially decreased the M1 macrophage density. Treatment with PTβR2I/Gel further reduced the M1 macrophage density observed at all three timepoints of wound healing. In addition to decreasing the number of M1 macrophages, the PTβR2I/Gel group also remarkably decreased the expressions of various pro-inflammatory cytokines, such as complement component 5a (C5a)^89^, granulocyte colony stimulating factor (G-CSF)^90^, interleukin-1 receptor antagonist (IL-1ra)^91^, and macrophage Inflammatory Protein-1 alpha (MIP-1α) (Fig. 5c, d)^92^. Notably, PTβR2I treatment significantly increased the density of M2 macrophages on day 3 compared to both the No treatment and Gel group (Supplementary Fig. 8). These results demonstrate that PTβR2I and the ROS-scavenging hydrogel can decrease inflammation during diabetic wound healing.

As diabetic wounds heal, the overproduced ROS aggravates inflammation and delays healing. We found that treating surgically induced wounds in diabetic mice with hydrogel (Gel group) significantly decreased the ROS content by days 3 and 8, the early and middle stages of healing (Fig. 5e, f), assumably due to the ROS scavenging property of the hydrogel. Adding PTβR2I into the hydrogel (PTβR2I/Gel group) did not change the wound ROS contents. By day 14 of the wound healing, the ROS content in both groups was reduced to an even lower level. Consistent with the ROS content results, the total antioxidant capacity was increased and the reactive nitrogen species (RNS) content was decreased in the wounds treated with Gel and PTβR2I/Gel (Fig. 5g, h). These results suggest that the developed wound dressing can decrease oxidative stress in diabetic wounds.

### Mechanisms of action of wound dressings in diabetic wounds

After finding that PTβR2I downregulated the TGFβ1/p38 pathway in vitro under high glucose, we then investigated whether the PTβR2I released from the wound dressings into diabetic wounds also down-regulated p38 signaling. On days 3 and 8, the expression of p-p38 was less pronounced in the PTβR2I/Gel group compared to the Gel group (Fig. 5i, j). These results are consistent with in vitro results, demonstrating that PTβR2I effectively downregulated the TGFβ1/p38 pathway leading to accelerated wound closure. We further evaluated the expression of p-Smad2/3 in the diabetic wounds on days 3 and 8 (Supplementary Fig. 9). At both time points, the PTβR2I treatment did not show an obvious effect on p-Smad2/3 expression compared to the Gel group, indicating that PTβR2I did not affect the TGFβ1/p-Smad2/3 pathway in the diabetic wounds.

### Effect of wound dressings on fibrosis in diabetic wounds

**Fig. 6:**
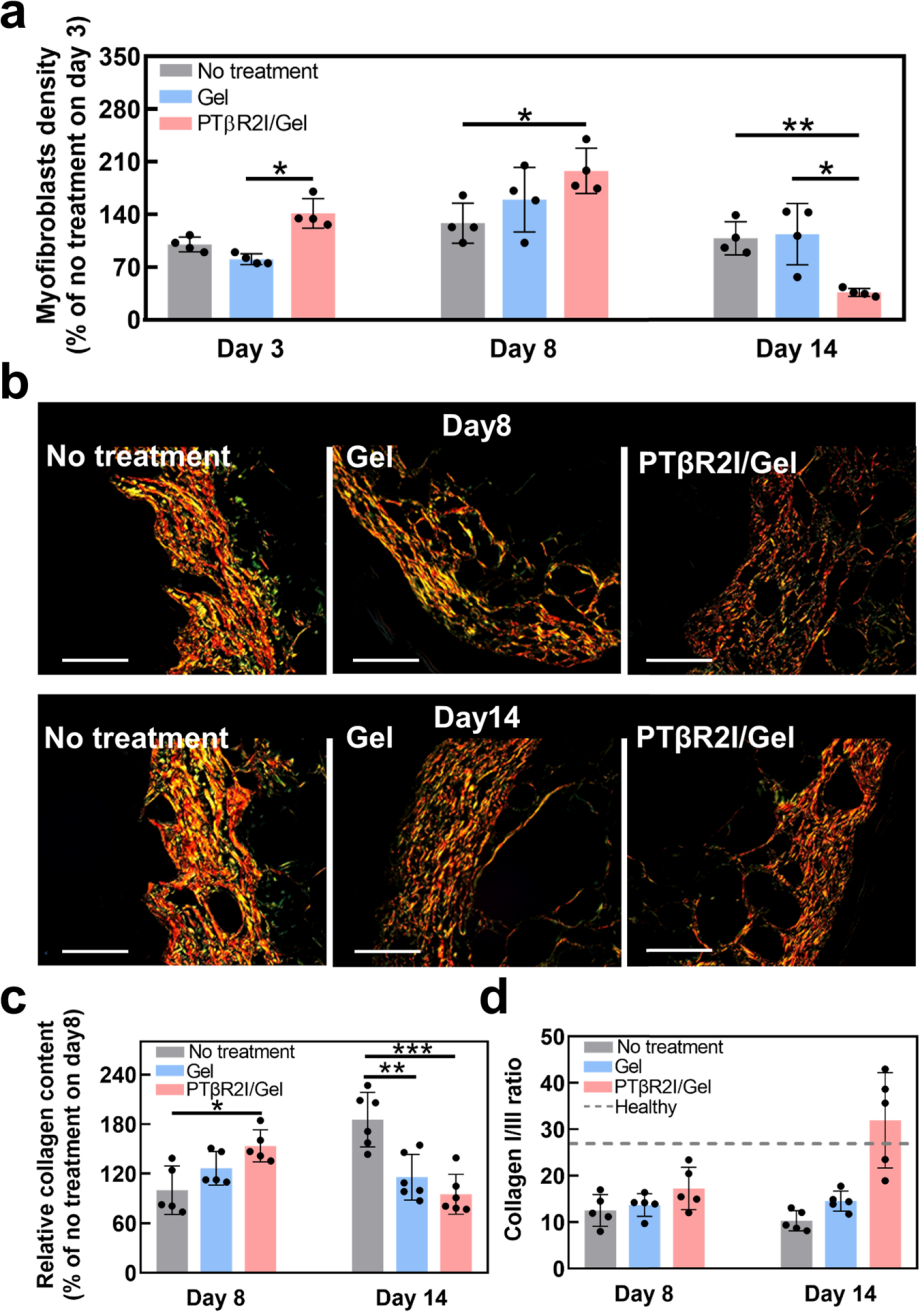
PTβR2I-releasing wound dressings prevented scar formation in diabetic wounds. **a** α-SMA^+^ and CD31^−^ myofibroblast densities (*n* = 4) quantified from images in Fig. 4a and images stained on day 3. Myofibroblasts are identified as α-SMA^+^ cells that are not colocalized with CD31^+^ endothelial cells. α-SMA^+^ cells that are colocalized with CD31^+^ endothelial cells are vascular smooth muscle cells/pericytes. **b** PSR staining study of wound sections at 8- and 14-days post- treatment. Scale bar = 50 µm. **c** Relative total collagen contents in the wound area on days 8 and 14, based on acquired PSR images (*n* ≥ 5). **d** Ratios of collagen I to collagen III at two time points, from PSR images (*n* = 5). The ratio for healthy skin is 26.3, indicated with a dashed line. All data demonstrated as mean ± standard deviation. Data were analyzed by one-way ANOVA with Bonferroni post-test (*p < 0.05, **p < 0.01, ***p < 0.001).

We next sought to determine whether the accelerated reepithelization in diabetic wounds treated with PTβR2I-releasing wound dressing was associated with scar formation. Because the expression of α-SMA is also a hallmark of myofibroblasts, we quantified the myofibroblast densities in our three experimental groups on days 3, 8, and 14 of healing (Fig. 6a). The continuing enhanced myofibroblast density of peptide groups in the early and middle healing stages indicated fast granulation tissue formation, in which myofibroblasts are the main producer and organizer of the ECM^93^. The reduced myofibroblast density in the PTβR2I/Gel group on day 14 reflected nearly complete wound contraction and tissue remodeling. However, significantly more myofibroblasts were found in the No-treatment group and the Gel group in the late stage of wound healing, which may instead lead to fibrosis and abnormal scarring.

In addition, picrosirius red staining (PSR) was performed at days 8 and 14 to reveal the tissue remodeling (Fig. 6b). On day 8, the PTβR2I/Gel group showed a significantly increased collagen content compared to the Gel alone and No-treatment groups (Fig. 6c), because collagen strengthens healing tissues in the middle, proliferative stage^94,95^. On the contrary, the collagen content decreased on day 14, likely due to less collagen deposition in the remodeling process in the PTβR2I/Gel group. At day 14, both the Gel and PTβR2I/Gel groups had COLAI/III ratios that were similar to that of unwounded tissue (Fig. 6d), suggesting the completion of collagen reformation during ECM remodeling.

### Effect of wound dressings on non-diabetic wounds

**Fig. 7:**
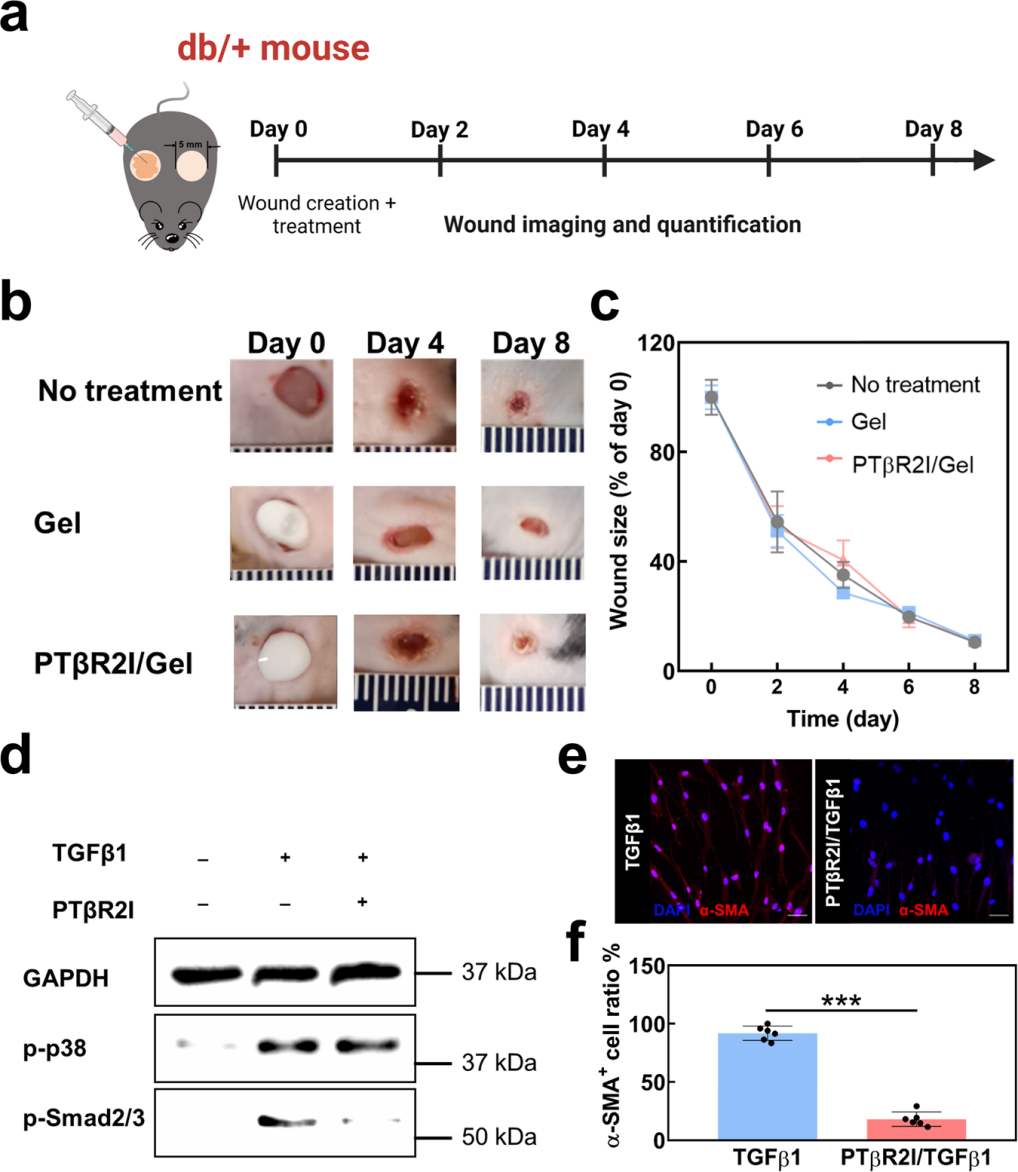
PTβR2I-releasing wound dressing treatment did not influence wound healing under low glucose conditions tested on *db/+* mice. **a** Timeline of *db/+* mice wound healing. **b** Representative images of wounds of *db/+* mice, taken from day 0 to day 8. Wounds were created using 5 mm biopsy punches on the dorsal skin of *db/+* mice and treated with gel and PTβR2I/Gel via subcutaneous injection. **c** Wound sizes over 8 days for each treatment. Wound size ratios were normalized to day 0 (*n* ≥ 4). **d** Immunoblotting analysis of p-p38 and p-Smad2/3 proteins in dermal fibroblasts cultured under low glucose conditions. GAPDH was used as a loading control. **e** Representative images of IF staining using α-SMA antibody of dermal fibroblasts cultured with 1 g L^−1^ glucose after 24 h. Scale bar = 50 µm. **f** Quantification of α-SMA^+^ cell ratio from the IF images (*n* = 6). All data demonstrated as mean ± standard deviation. Data were analyzed by one-way ANOVA with Bonferroni post-test (n.s.*p* > 0.05, ***p < 0.001).

Interestingly, PTβR2I was not effective on genetically mutated *db/+* mice. We performed the same wound assay on *db/+* mice and monitored the wound closure for eight days (Fig. 7a). The No-treatment, Gel, and PTβR2I/Gel groups all had similar healing rates, with wound sizes less than 10% of the original after 8 days (Fig. 7b, c). To delineate the mechanism, we performed in vitro immunoblotting on dermal fibroblasts under normal glucose conditions that mimicked the microenvironment of *db/+* mice (Fig. 7d). PTβR2I apparently inhibited the TGFβ1/p-Smad2/3, but did not downregulate p-p38, aligning with in vivo results. The IF study further validated that PTβR2I suppressed myofibroblasts activation under normal glucose conditions (Fig. 7e, f).

Overall, PTβR2I appeared to inhibit the TGFβ1/p-Smad2/3 pathway under normal glucose conditions while having no substantial inhibition effect when the glucose level was as high as that found in diabetes. Under high glucose conditions, PTβR2I inhibited the TGFβ1/p38 pathway leading to accelerated wound healing in *db/db* mice.

## Discussion

Impaired wound healing is a lifetime risk for patients with diabetes. Various therapies have been developed to heal diabetic wounds, yet effective treatment remains a challenge, largely because current therapies cannot efficiently address the key intrinsic causes of slow diabetic wound healing, i.e., abnormal skin cell functions (particularly migration), delayed angiogenesis, and chronic inflammation. TGFβ1/p38 signaling is directly associated with these key intrinsic causes^56,96,97^. The phosphorylation of p38 is upregulated in the wounds of *db/db* mice with sustained hyperglycemia^56^. Using TGFβ inhibitors or anti-TGFβ antibodies to decrease the amount of active TGFβ1 in the wounds can reduce its deteriorate effect^50–52^. However, it is challenging to inhibit only the TGFβ1/p38 pathway and not interfere with other TGFβ1 signaling axes that are essential for wound healing, such as the TGFβ1/Smad2/3 pathway^98,99^. To the best of our knowledge, no existing approach has addressed this challenge.

In this work, we show that wound dressings consisting of PTβR2I and a ROS-scavenging hydrogel accelerated wound healing in *db/db* mice. We demonstrated that PTβR2I binds with TβR2 and differentially regulates TGFβ1 pathways: PTβR2I downregulates the TGFβ1/p38 pathway while not affecting the TGFβ1/Smad-2/3 pathway under the hyperglycemia environment in diabetic wounds. We found that PTβR2I enhanced the migration of keratinocytes, dermal fibroblasts, and endothelial cells; promoted cell proliferation and paracrine effects; facilitated endothelial morphogenesis; and decreased proinflammatory cytokine expression in skin cells under TGFβ1 and high glucose conditions (Fig. 1j–u). We used an injectable, thermosensitive, fast-gelling, and ROS-scavenging hydrogel as a wound dressing to encapsulate PTβR2I and continuously release the peptide to the wounds. Many hydrogels have been used as wound dressings, such as gelatin^100^, hyaluronic acid^101^, alginate^102^, and chitosan^103^, poly(vinyl alcohol)^104^, poly(ethylene glycol)^105^, and polypeptides^106^. Compared to these hydrogels, our hydrogel has unique properties such as thermosensitivity, fast gelation, and ROS scavenging. The thermosensitivity and fast-gelling properties allowed the PTβR2I to retain in the wounds after administration (Fig. 2c). The ROS-scavenging property enabled the wound dressing to capture the upregulated ROS in diabetic wounds (Figs. 2a, e, f and 5e–h). The PTβR2I-releasing wound dressing significantly accelerated reepithelization, promoted host cell proliferation and vessel formation, decreased inflammation and oxidative stress, and regulated collagen deposition without forming scar tissues. Intriguingly, under euglycemia, PTβR2I inhibited the TGFβ1/Smad-2/3 pathway without affecting the TGFβ1/p38 pathway, resulting in no improvement of wound healing when tested in *db/+* mice (Fig. 7d).

Keratinocytes, fibroblasts, and endothelial cells are respectively responsible for epithelialization, wound contraction, and angiogenesis during wound healing. In diabetic wounds, the TGFβ1 and high glucose conditions impair the migration of these cells, leading to delayed epithelialization, wound contraction, and angiogenesis. Interestingly, we found that PTβR2I enhanced the migration of keratinocytes, fibroblasts, and endothelial cells under TGFβ1 and high glucose conditions (Fig. 1m–o). Following cell recruitment, cell proliferation also plays an important role in wound healing, especially for larger and chronic diabetic wounds where migration alone is insufficient for wound closure. We demonstrated that PTβR2I increased the proliferation of keratinocytes, fibroblasts, and endothelial cells. Notably, PTβR2I also stimulated endothelial cells to form lumens (Fig. 1p, q), and upregulated the expressions of *VEGFA* and *HGF* in endothelial cells (Fig. 1r). These factors not only promote angiogenesis but also positively affect the migration, proliferation, and differentiation of keratinocytes^82^. As a result, the diabetic wounds treated with PTβR2I-releasing wound dressings had significantly greater vessel density than those dressed without PTβR2I (Fig. 4a, b). The PTβR2I released from the wound dressings favorably influenced the migration of keratinocytes throughout the inflammatory, proliferative, and remodeling phases of wound healing (Fig. 3d). In addition, more K14^+^ keratinocytes in the basal layer underwent differentiation into K10^+^ keratinocytes in the suprabasal layers^83,107^.

Chronic inflammation and high oxidative stress critically delay diabetic wound healing. We demonstrated that PTβR2I suppressed the expression of proinflammatory cytokines such as *TNFα*, *IL1B*, and *IL6* in keratinocytes, fibroblasts, and macrophages in vitro (Fig. 1s–u). This result is consistent with previous reports that inhibiting the p38 pathway reduced the expression of pro-inflammatory cytokines in skin cells^56,108,109^. In diabetic wounds, PTβR2I released from the wound dressings decreased the M1 macrophage density at all three stages of the healing (Fig. 4a, b). Overproduced ROS in diabetic wounds can contribute to chronic inflammation. We showed that ROS-scavenging hydrogel in the wound dressing can scavenge ROS and continuously mitigate oxidative stress.

Diabetic wounds are characterized by impaired production of ECM, a crucial facilitator of wound healing, from the inflammatory phase through the remodeling phase^110,111^. Collagen, the most abundant ECM in the skin, is significantly under-produced in diabetic wounds^112,113^. In addition, the ratio of the two major collagen types (I and III) is abnormally different from that of uninjured skin^114–116^. Myofibroblasts are primarily responsible for collagen deposition in wounds. During normal wound healing, the number of myofibroblasts decreases dynamically in the final remodeling stage to avoid excessive collagen deposition that causes scars. For these reasons, it is important to regulate myofibroblasts to achieve normal deposition of collagen and a normal collagen I/III ratio. Our work demonstrated that PTβR2I-releasing wound dressings beneficially modulated the number of myofibroblasts in the progression from the inflammatory phase to the remodeling phase (Fig. 6a), most probably because PTβR2I did not interfere with the TGFβ1/Smad2/3 pathway. Collagen deposition was increased during the inflammatory and proliferative phases, and decreased during the remodeling phase without inducing scar formation. In addition, the collagen I/III ratio in the remodeling phase was similar to that of uninjured tissue.

In this work, db/db mice were used to evaluate the therapeutic efficacy of developed wound dressings. Several previously studies utilized streptozotocin (STZ) and high-fat diet-induced mice for diabetic wound healing^117–119^. Compared to these models, db/db mice exhibit characteristics such as morbid obesity and chronic hyperglycemia, making them more suitable for evaluating the therapeutic efficacy of wound dressings under type II diabetic conditions.

There are some limitations in this work. The therapeutic efficacy was tested on wounds of db/db mice, which may not be representative of the pathophysiology of human diabetic wounds. Future studies will use large animals to improve the translation potential of the developed wound dressing. In addition, the hydrogel concentration and injection dosage need to be optimized for different animal models. Despite these limitations, the current wound dressing presents an effective approach to accelerate diabetic wound healing.

**Fig. 8:**
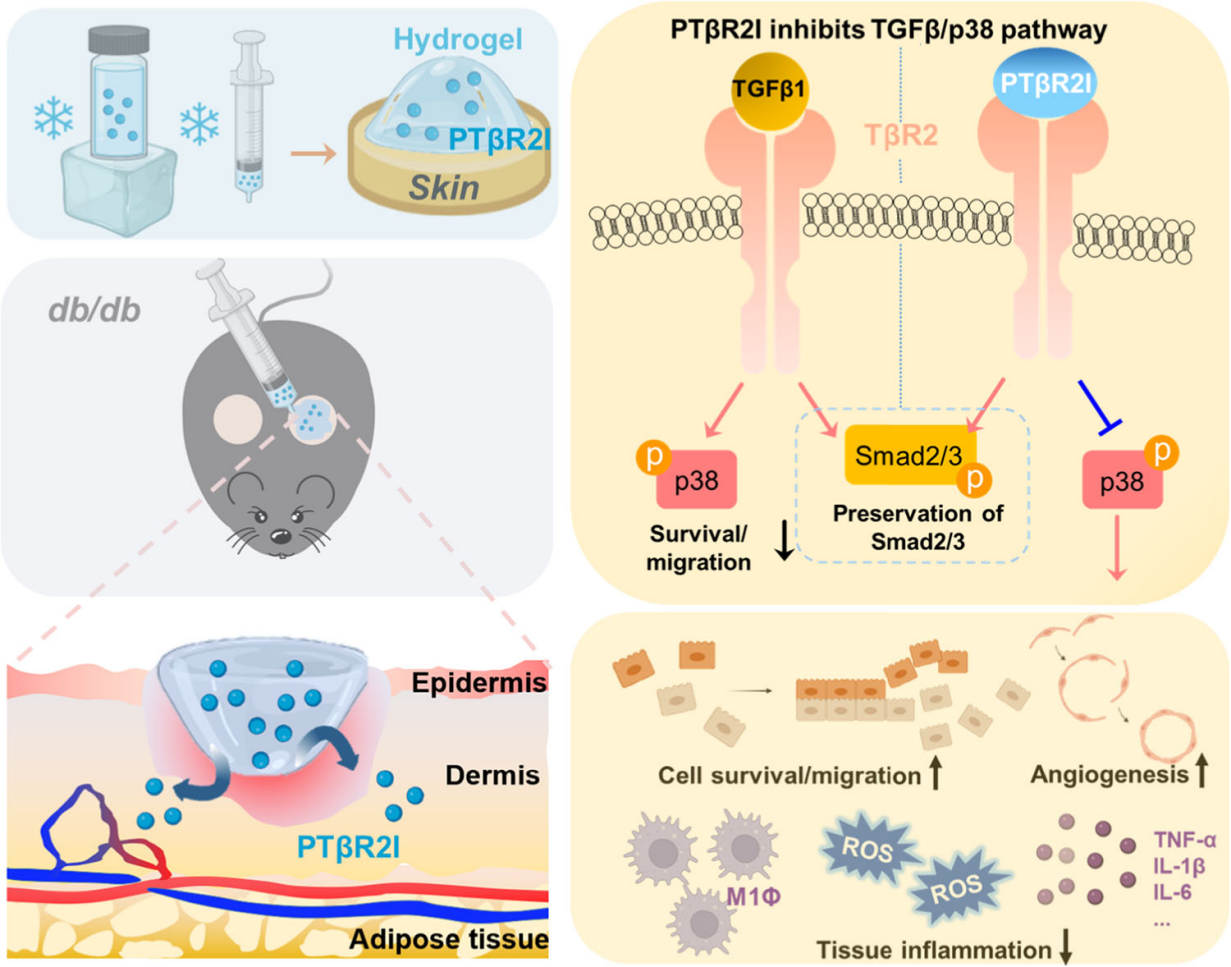
Mechanisms of accelerated diabetic wound healing achieved by using wound dressing containing PTβR2I and ROS-scavenging hydrogel. The PTβR2I gradually released from the wound dressing adaptively regulated TGFβ pathways by inhibiting p38 signaling while not interfering with Smad2/3 signaling. The hydrogel scavenged upregulated ROS in the wounds. As a result, the wound dressing promoted the survival and migration of skin cells, enhanced angiogenesis, and decreased tissue inflammation and oxidative stress.

In summation, we report that wound dressings consisting of PTβR2I and a ROS-scavenging hydrogel accelerated diabetic wound healing, by adaptively regulating the TGFβ1/p38 and the TGFβ1/Smad2/3 pathways (Fig. 8). The wound dressings performed multiple functions: stimulating skin cell migration, proliferation, and paracrine effects; promoting endothelial morphogenesis and angiogenesis; and reducing tissue inflammation and oxidative stress under TGFβ1 and high glucose conditions. These wound dressings more quickly accelerated wound closure than using growth factors^120,121^, protein^122^, exogenous cells^123^, or oxygen therapy in the same animal model^73^. A limitation of the current study is that the rodent models do not fully mimic the complex human pathophysiology, and implications of microvascular occlusive disease that may occur in the setting of chronic diabetes. In future studies, we will test the developed wound dressings in large animals (e.g., pigs with and without diabetes), or in models with concomitant hyperlipidemia and vascular occlusive disease.

## Methods

### Materials

All chemicals and bioreagents were purchased from Millipore-Sigma unless otherwise stated. N-isopropylacrylamide (NIPAAm, TCI) was recrystallized three times before use. Hydroxyethyl methacrylate (HEMA, Alfa Aesar) was used after passing it through an inhibitor removal column. PTβR2I was synthesized by Celtek Bioscience based on the sequence provided. Acryloyl chloride, 4-(hydroxymethyl)-phenylboronic acid pinacol ester (HPPE), acrylic acid (AAc), and benzoyl peroxide (BPO, Life Technologies) were used as received.

### Binding of PTβR2I to TβR2, TβR1, and IgG

To test the ability of PTβR2I to bind to TβR2, a binding assay was performed as previously described^124^. TβR1, TβR3, and IgG were used as controls. In brief, ELISA plates (Costar) were coated with 20 nM of TβR2 (R&D), TβR1 (R&D), or IgG (R&D) and incubated at 4 °C overnight. The plate was blocked with Tris-buffered Saline (TBS) containing 5% bovine serum albumin (BSA) for 1 h. Then PTβR2I (0.5 µg mL^−1^) was added to the plate and incubated for 2 h. The fluorescence intensity was read by a microplate reader (Molecular Devices) with a predefined wavelength (excitation/emission = 485/535 nm).

To test PTβR2I binding to cells, HDFs (Lonza) were seeded on type I collagen-coated cover glasses. After 24 h, the following treatments were applied: 1) PTβR2I solution (10 µg mL^−1^) for 48 h; 2) PTβR2I solution (10 µg mL^−1^) for 24 h, and then TGFβ1 (10 ng mL^−1^) for another 24 h; 3) TGFβ1 (10 ng mL^−1^) for 24 h, and then PTβR2I (10 µg mL^−1^) for another 24 h; and 4) both PTβR2I (10 µg mL^−1^) and TGFβ1 (10 ng mL^−1^) for 48 h. HDFs cultured without PTβR2I or TGFβ1 for 48 h served as the control group. After the treatments, the supernatant in the groups added with TGFβ1 was collected, and the unbound TGFβ1 was measured using a TGFβ1 ELISA kit (Thermofisher). The cells were fixed by 4% paraformaldehyde and blocked with 10% goat serum in DPBS. Primary antibody mouse anti-TβR2 (1:300, Santa Cruz) that was diluted in DPBS containing 3% BSA was incubated with the cells overnight at 4^o^C. The cells were washed three times with DPBS. A secondary antibody Alexa Fluro-647 (1:300, Fisher Scientific) was then applied for 2 h at room temperature, followed by incubating with DAPI for five minutes. Fluorescent images were taken by a confocal microscope (Olympus FV1200).

To validate the above results, the fluorescence intensity of PTβR2I bound to HDFs was measured for each treatment group. Briefly, HDFs (Lonza) were seeded on type I collagen-coated 96-well black microplates. After 24 h, the treatments described above were applied. Cells were washed three times with DPBS to remove free PTβR2I. The fluorescence intensity was measured using the same microplate reader.

### Cell culture

HDFs were cultured using Gibco Dulbecco’s modified Eagle’s medium (DMEM, Gibco) supplemented with 10% fetal bovine serum (FBS, Atlanta Biologicals) and 1% penicillin-streptomycin (P/S). Human keratinocytes HaCaT cells were purchased from AddexBio and cultured in AddexBio-Optimized DMEM containing 10% FBS and 1% P/S. Human arterial endothelial cells (HAEC) were obtained from Cell Systems and cultured in a complete medium kit supplemented with serum (FBS, 10%), and culture boost. All cell types were incubated at 37^o^C with 5% CO_2_ until reaching 80–90% confluency before passaging. The culture medium was replenished every other day.

Macrophages were derived from THP-1, a human monocytic cell line (ATCC). THP-1 cells were treated with 100 ng/mL phorbol 12-myristate 13-acetate (PMA, Millipore-Sigma) for 48 h, followed by 24 h in RPMI 1640 medium (ATCC) with 10% FBS and 1% P/S. To induce differentiation into M1 macrophages, the cells were treated with 100 pg mL^−1^ lipopolysaccharide (LPS, Millipore-Sigma) and 20 ng mL^−1^ interferon-gamma (IFN-γ, Millipore-Sigma) for 48 h.

### Peptide cytotoxicity test

HDFs, HaCaTs, and HAECs were seeded in 96-well plates at densities of 1.5 × 10^4 ^cells mL^−1^, 2 × 10^4^ cells mL^−1^, and 2 × 10^4^ cells mL^−1^, respectively. To investigate the effect of PTβR2I on cell viability, different concentrations of PTβR2I ranging from 0 to 100 µg/mL were added to the culture medium. After 24 h of incubation, cell viability was assessed using MTT assay^125,126^.

### Cell proliferation under high glucose and TGFβ1 conditions

HDFs, HaCaTs, and HAECs were seeded in 96-well plates at respective densities of 1.5 × 10^4^ cells mL^−1^, 2 × 10^4^ cells mL^−1^, and 2 × 10^4^ cells mL^−1^. High glucose (4.5 g L^−1^) basal medium with 1% P/S was used for the culture. After 24 h, TGFβ1 (10 ng mL^−1^) or TGFβ1 (10 ng mL^−1^)/PTβR2I (10 µg mL^−1^) was added to the medium. The culture continued for three days. Cells were then treated with papain solution and incubated at 60 °C for 24 h. The dsDNA content was tested using a Quant-iT™ PicoGreen dsDNA Assay Kit (Invitrogen)^73,127,128^.

### Cell migration under high glucose and TGFβ1 conditions

HDFs, HaCaTs, and HAECs were cultured in 6-well plates using serum-free high glucose (4.5 g L^−1^) basal medium with 1% P/S. After the cells reached confluency, a 200-µL pipet tip was used to scrape the cell monolayer. Then 3 mL of medium containing TGFβ1 (10 ng mL^−1^) or TGFβ1 (10 ng mL^−1^)/PTβR2I (10 µg mL^−1^) was added to the wells. The cells were imaged at predetermined time points using a bright-field microscope (Olympus IX70). The distances between the scratch walls were measured using ImageJ to calculate the migration ratio^72,73,127^.

### Endothelial cell lumen formation

A 3D collagen gel model was used to evaluate endothelial cell lumen formation under high glucose and TGFβ1 conditions^73,127^. Briefly, the collagen gel was formed by mixing 4 mg mL^−1^ of rat tail type I collagen solution (Life Technologies), FBS, DMEM, and NaOH. Then 500 µL of the mixture was transferred to a 48-well plate and placed in a 37 °C incubator for 30 min to allow gelation. HAECs were then seeded into the collagen gel at a density of 2 × 10^4^ cells/well. TGFβ1 (10 ng mL^−1^) or TGFβ1 (10 ng mL^−1^)/PTβR2I (10 µg mL^−1^) was then added. After three days of culturing, cells were stained with F-actin (Abcam) and DAPI. The constructs were imaged by Olympus FV1200 confocal microscopy with z-stack mode. The lumen density was calculated from the images^73,127^.

### Synthesis and characterization of hydrogel

The ROS-responsive monomer 4-(acryloxymethyl)-phenylboronic acid pinacol ester (AHPPE) was synthesized by acrylation of HPPE following a previously established method^73^. ^1^H-NMR was used to verify the chemical structure (-*CH*_*2*_= at 6.36 ppm and 5.77 ppm, =*CH*- at 6.09 ppm, *O-CH*_*2*_- at 5.13 ppm, -*C*_*2*_*H*_*6*_*-B* at 7.74 ppm and 7.29 ppm, and -*CH*_*3*_ at 1.26–1.55 ppm). The hydrogel was synthesized by free radical polymerization of NIPAAm, HEMA, and AHPPE using BPO as an initiator^129,130^. The molar feed ratio of the three monomers was 75/15/10. The real monomeric ratio of the polymer was calculated from ^1^H-NMR. After AHPPE is completely cleaved by ROS, the final product becomes poly (NIPAAm-*co*-HEMA-*co*-acrylic acid (AAc)). We synthesized this polymer by free radical polymerization using BPO as an initiator^129,130^. The molar feed ratio of NIPAAm/HEMA/AAc was 75/15/10. In addition, a control, non-ROS responsive hydrogel was synthesized by free radical polymerization of NIPAAm, HEMA, and acrylate-polylactide at a ratio of 75/15/10 using our previously established approach^83^.

The hydrogel solution of 6 wt% was prepared by dissolving the polymer into DPBS with continuous stirring at 4 °C for 12 h. The solution was then kept on ice until use. The injectability of the hydrogel solution was examined at 4 °C and 12 °C, using a 27 G needle^73,131^. The gelation time of the 4 °C solution was evaluated at 30 °C and 37 °C, as described previously^131^.

To measure water content, the hydrogel was first immersed in DPBS at 37 °C for 5 h. The gel was then taken out and the wet weight was measured as w_1_. Then the hydrogel was freeze-dried, and the dry weight was measured as w_2_. The water content was calculated as (w_1_-w_2_)/w_2 _× 100%^131^.

The gelation temperature of the hydrogel was evaluated by rheological test using a Discovery HR-20 rheometer. The geometry was 20 mm, and the gap was 500 µm. The oscillation frequency and strain were 1 Hz and 2%, respectively. Temperature swept from 10 °C to 30 °C at a rate of 2 °C/min during the test.

To test the cytotoxicity of the degradation product, poly (NIPAAm-*co*-HEMA-*co*-AAc) (Fig. 3a**)** was dissolved in DMEM with 10% FBS at different concentrations. HDFs were seeded at a density of 3×10^4^ cells mL^−1^ in a 96-well plate. After 24 h, the cells were treated with the medium containing the degradation product, and after 48 h, the cell viability was quantified by MTT assay^125,132^. The medium without degradation product was used as a control.

To test the ROS responsiveness of the hydrogel, 200 µL of hydrogel solution was added to a 1.7 mL microcentrifuge tube. After gelation at 37 °C, the supernatant was taken out, and replaced with 200 µL of DPBS with or without 1 mM H_2_O_2_. The non-ROS responsive hydrogel was used as a control. The degradation was conducted at 37 °C for 14 days. At each time point, the samples were freeze-dried and the remaining weight was measured.

To test total antioxidant capacity, the hydrogel was cast in a 96-well plate. 200 µL of DPBS with or without 100 µM H_2_O_2_ was then added into the wells. After incubation for 48 h, the supernatant was collected. The total antioxidant capacity was measured using an antioxidant assay kit (Millipore-Sigma, catalog# MAK334) following the manufacturer’s instructions.

### Hydrogel and PTβR2I retention in diabetic wounds

To evaluate the retention of hydrogel and PTβR2I in diabetic wounds, PTβR2I (FITC-labeled) was mixed separately with the hydrogel solution and the collagen solution. The collagen solution was used as a control. All animal experiments were performed in accordance with the National Institutes of Health Guide for the Care and Use of Laboratory Animals. The protocol was approved by the Institutional Animal Care and Use Committee of Washington University in St. Louis. Female BKS.Cg-Dock7^m^ + /+ Lepr^db/J^ mice (*db/db* mice, Jackson Laboratories) aged eight weeks were used. The mice were anesthetized by isoflurane inhalation, and an electronic shaver and hair removal cream was used to thoroughly remove the hair from the dorsal skin. Before surgery, ethanol pads and betadine were applied in series on the dorsal skin. Then, with a biopsy punch, two symmetric 5-mm diameter wounds were made on the dorsal skin of each mouse. Each wound was then dressed with either the PTβR2I/hydrogel mixture or the PTβR2I/collagen mixture. After 24 h, the mice were sacrificed and the wounds were collected, followed by imaging using an in vivo imaging system (IVIS Spectrum, Perkin Elmer) with an excitation filter of 485 nm and an emission filter of 535 nm. The fluorescence images were quantified by Living Image software (PerkinElmer Inc.)^125,131^.

### Subcutaneous implantation of hydrogel

All animal care and experiment procedures were conducted in accordance with the National Institutes of Health guidelines. The animal protocol was approved by the Institutional Animal Care and Use Committee of Washington University in St. Louis. To examine the in vivo toxicity of the hydrogel, 6 wt% hydrogel solution was subcutaneously injected into the 8-week-old C57BL/6 J mice. Prior to the injection, the pre-cooled hydrogel solution was sterilized under UV light for 30 min. As controls, mice injected with collagen gel were used. After seven days, tissue specimens were harvested at the injection sites and fixed with 4% paraformaldehyde for 24 h. The tissue sections (5 µm thick) were stained with anti-F4/80 antibody (Santa Cruz) and DAPI. The stained sections were imaged with an Olympus FV1200 confocal microscope. The F4/80+ cell ratio was quantified by normalizing F4/80+ cell number to the total cell number in each image.

### Wound dressing fabrication and PTβR2I release kinetics

Wound dressings were fabricated by encapsulating PTβR2I in the hydrogel solution at 4 °C. Briefly, PTβR2I solution was mixed with 6 wt% hydrogel solution to reach final PTβR2I concentrations of 10 µg/mL, 20 µg/mL, and 50 µg/mL. The mixtures were stirred at 4 °C for 12 h. To determine PTβR2I release kinetics, the mixtures were first incubated at 37 °C to induce gelation. After 1 h, the supernatant was replaced by a release medium (DPBS with 1% P/S). The medium was then collected at pre-determined time points for 21 days, and a fresh release medium was added after each collection. The concentration of the released PTβR2I was determined by measuring the fluorescence intensity and a standard curve. The bioactivity of the released PTβR2I was evaluated by its ability to bind to the TβR2 on the HDFs. The collected released medium was added to the culture medium of HDFs that were seeded in a 96-well plate. The controls were the same concentrations of fresh PTβR2I solution as those released from the hydrogel at certain time points. After 2 h of incubation, the cells were washed three times with DPBS, and the fluorescence intensity was measured using a microplate reader (excitation/emission = 485/535 nm). The bioactivity was quantified by normalizing the intensity of the released PTβR2I to that of the corresponding control.

### mRNA expression for in vitro cultured cells

To determine the mRNA expression of the cells (HDFs, HaCaTs, and HAECs) treated with TGFβ1 (10 ng mL^−1^) or TGFβ1 (10 ng mL^−1^)/PTβR2I (10 µg mL^−1^) under high glucose conditions, RNA was extracted using Trizol (Invitrogen) and reverse transcribed using a cDNA synthesis kit (Applied Biosystems). Gene expression was performed by real-time RT-PCR, using Maxima SYBR Green/Fluorescein Master Mix (Thermofisher) and selected primer pairs (Supplementary Table 1). β-actin served as the housekeeping gene. The ΔΔCt method was used for data analysis^73,127^.

### Protein array assay

Wounded tissues were collected and lysed at predetermined timepoints. The protein concentrations were quantified by Bradford assay. The samples were tested using a Proteome Profiler Mouse Angiogenesis Array kit (R&D Systems) and a Mouse Cytokine Array kit (R&D Systems). The intensities of the dots on the membranes in the kits were quantified using Image Lab software^73^.

### Implantation of wound dressings into diabetic wounds

Eight-week-old, female *db/db* mice were used. Carprofen tablets (1/4 tablet per mouse) were given orally 48 h before surgery and continued for seven days after the surgery as an analgesic. One day before surgery, the blood glucose levels of the mice were tested with a glucometer to confirm they were above 300 mg/dL. The mice were anesthetized by isoflurane inhalation, and the dorsal surface was thoroughly shaved. Then a 5-mm diameter biopsy punch (VWR) was used to create two symmetric full-thickness wounds on the back of each mouse. Next, 100 µL of liquid wound dressing, with or without 50 µg/mL of PTβR2I, was applied topically to the wound sites. The mice were observed every other day, and digital photographs of the wounds, with a ruler at the side, were acquired by a digital camera. Wound sizes were calculated using Image J.

### In vivo oxidative stress measurement

On day 3, skin tissues at wound sites for each group were harvested, weighed, minced into small pieces, and homogenized in DPBS/protease inhibitor on ice. The supernatant was collected. The samples were then tested for the total antioxidant capacity and RNS content using an antioxidant assay kit (Millipore-Sigma) and a mouse RNS ELISA kit (MyBiosource), respectively.

### Western blot analysis

For in vitro tests, protein lysates were collected from HDFs. Pre-cooled cell scrapers were used to detach the cells, and the cells were re-suspended in RIPA lysis buffer with the inhibitor cocktail. For in vivo tests, wound skin tissues were washed three times with pre-cooled DPBS, dissected into small pieces, and re-suspended in RIPA lysis buffer containing protease and phosphatase inhibitor. Homogenization was then performed thereafter with an ultrasonic processor (Cole Parmer). After centrifugation at 12,000 g for 20 min, the supernatants were collected, and protein concentrations were measured using a Bradford protein assay kit (Bio-rad).

The protein samples were separated by 10% Mini-PROTEAN TGX stain-free precast gels (Bio-rad) and transferred onto immune-blot PVDF membrane. The blots were washed three times with DPBS containing 0.1% Tween 20 (PBST), blocked with 5% milk powder in PBST buffer for 40 min, and incubated with primary antibodies at specific dilutions overnight at 4 °C. The primary antibodies included rabbit GAPDH antibody (1:5000, Cell signaling, Cat#2118), rabbit anti-α-smooth muscle actin (α-SMA) antibody (1:2000, Cell signaling, Cat#19245), rabbit anti-phospho-Smad2(Ser465/467)/Smad3 (Ser423/425) (1:500, Cell signaling, Cat#8828), and rabbit anti-phospho-p38 (1:500, Cell signaling, Cat#4511). The membranes were washed three times with PBST and incubated with horseradish peroxidase (HRP)-conjugated secondary antibodies (1:2500, Abcam, Cat#ab205718). Immunoblots were then washed with PBST and detected with a WesternBright HRP substrate detection kit (Advansta). The ChemiDoc XPS^+^ system (Bio-rad) was used to image the blots. All blots were processed in parallel and were derived from the same experiment. The uncropped scans of the blots are presented in Supplementary Fig. 10.

### Histological and immunofluorescence analyses

Wound tissues were collected on days 3, 8, and 14 after administration of wound dressings. The tissues were fixed in 4% paraformaldehyde for 24 h. Then the samples with the whole wound areas were cross-sectioned into 5 µm thick slices. Masson’s Trichrome staining (MTS), and picosirius red staining were performed. The epidermal thickness was calculated from the MTS images in the wounded region. For immunohistochemical staining, tissue sections were stained with primary antibodies including mouse anti-cytokeratin 14 (1:1000, abcam, Cat# ab7800), rabbit anti-cytokeratin 10 (1:3000, abcam, Cat# ab76318), rabbit anti-CD31 (1:50, abcam, Cat# ab28364), mouse anti-α-SMA (1:10000, abcam, Cat# ab7817) and rat anti-Ki67 (1:100, Thermofisher, Cat# MA5-14520), rabbit anti-CD86 (1:50, Cell Signaling, Cat#91882), CellROX deep red (1:500, Thermofisher, Cat#C10422), and incubated at 4 °C overnight. Alexa 647 goat anti-rabbit (1:300, Thermofisher, Cat#A-21245), Alexa 546 goat anti-mouse (1:300, Thermofisher, Cat#A-11003), Alexa 488 goat anti-rabbit secondary antibodies (1:300, Thermofisher, Cat#A-11034) were then applied. DAPI (Millipore-Sigma) was used to stain the nuclei. Images were acquired by Olympus confocal microscope and analyzed using ImageJ.

### Statistical analysis and reproducibility

Data are presented as mean ± standard deviation unless otherwise stated. Statistical analysis was performed using one-way or two-way ANOVA followed by the Bonferroni post-test, using GraphPad Prism 8. Significance was defined as *P* < 0.05.

### Reporting summary

Further information on research design is available in the Nature Research Reporting Summary linked to this article.

## Supplementary information

SUPPLEMENTAL information

reporting summary

## Data Availability

The data in the current study are available upon reasonable request.
