# Supplementary material for: Thermosensitive and antioxidant wound dressings capable of adaptively regulating TGFβ pathways promote diabetic wound healing
Source: NPJ Regen Med. 2023 Jul 8;8:32. doi: 10.1038/s41536-023-00313-3 (PMC10329719; doi:10.1038/s41536-023-00313-3)
Supplement: Supplementary file 1 — SUPPLEMENTAL information [file 41536_2023_313_MOESM1_ESM.pdf]

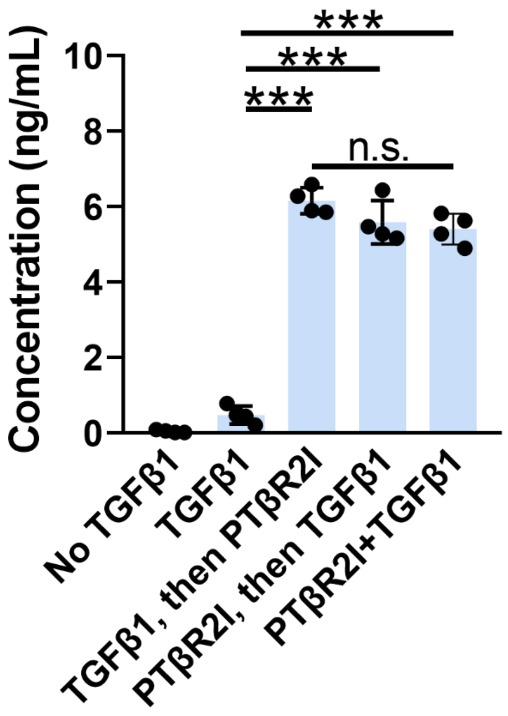

Supplementary Figure 1. Concentration of unbound TGFβ1 in the supernatant during binding test examined using a human/mouse TGFβ1 ELISA kit.

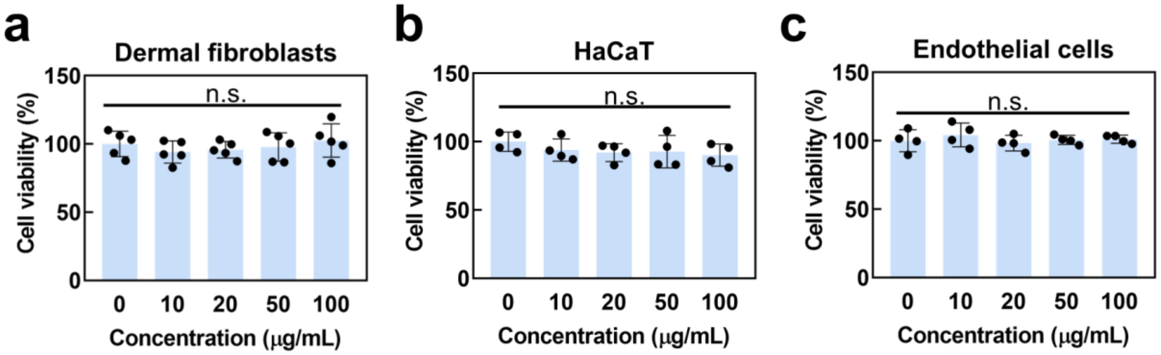

Supplementary Figure 2. Cytotoxicity test of PTβR2I on major skin cells, including (**a**) dermal fibroblasts (n=5), (**b**) HaCaT (n=4), and (**c**) endothelial cells (n=4). Data were analyzed by one-way ANOVA with Bonferroni post-test ($^{n.s.}p>0.05$).

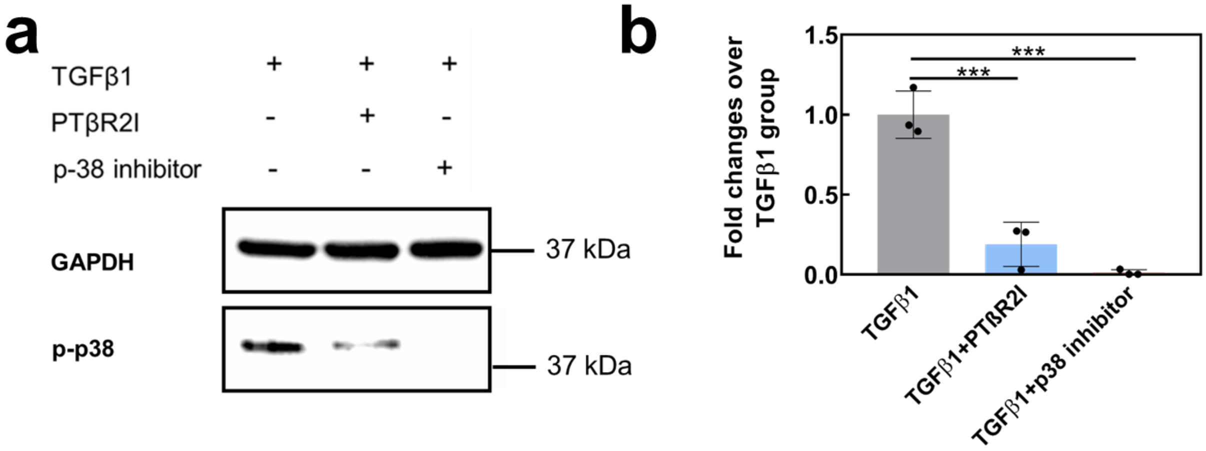

Supplementary Figure 3. Analysis of p-p38 expression in dermal fibroblasts after treating the cells with TGFβ1 alone, TGFβ1 with PTβR2I, or TGFβ1 with p-38 inhibitor. **a.** Immunoblotting of p-p38 in dermal fibroblasts after different treatments. GAPDH was loading control. **b.** Quantification of fold change of p-p38 expression after different treatments (n=3, ***p<0.001).

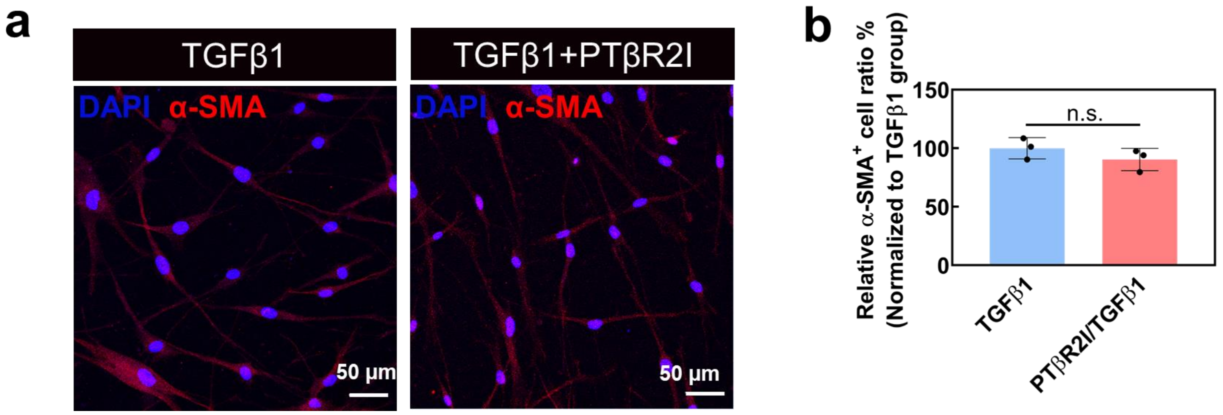

Supplementary Figure 4. Transition of fibroblasts to myofibroblasts under high glucose condition after treating with TGFβ1 or TGFβ1/PTβR2I. **a.** Representative images of cells stained for α-SMA and DAPI. The cells were cultured on 2D collagen gels. **b.** Quantification of ratio of α-SMA+ myofibroblasts.

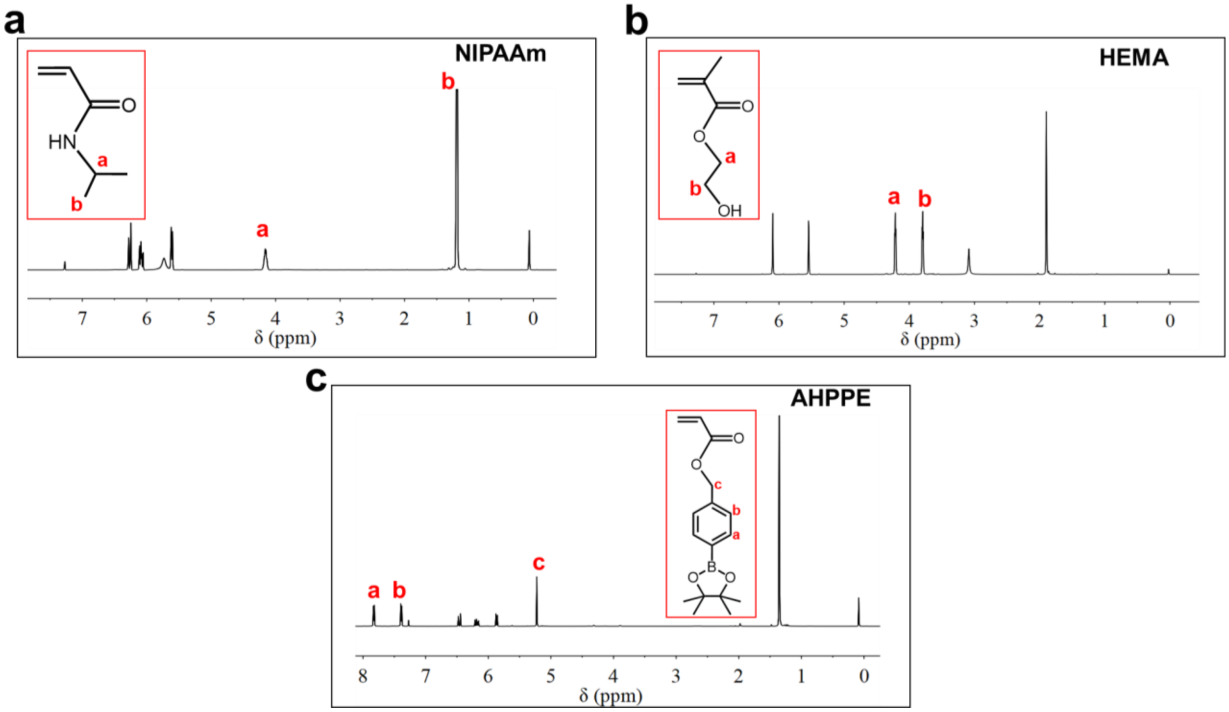

Supplementary Figure 5. ^{1}H-NMR spectrum of the monomers (**a**) NIPAAm, (**b**) HEMA, (**c**) AHPPE, and its corresponding characteristic peaks in the polymer.

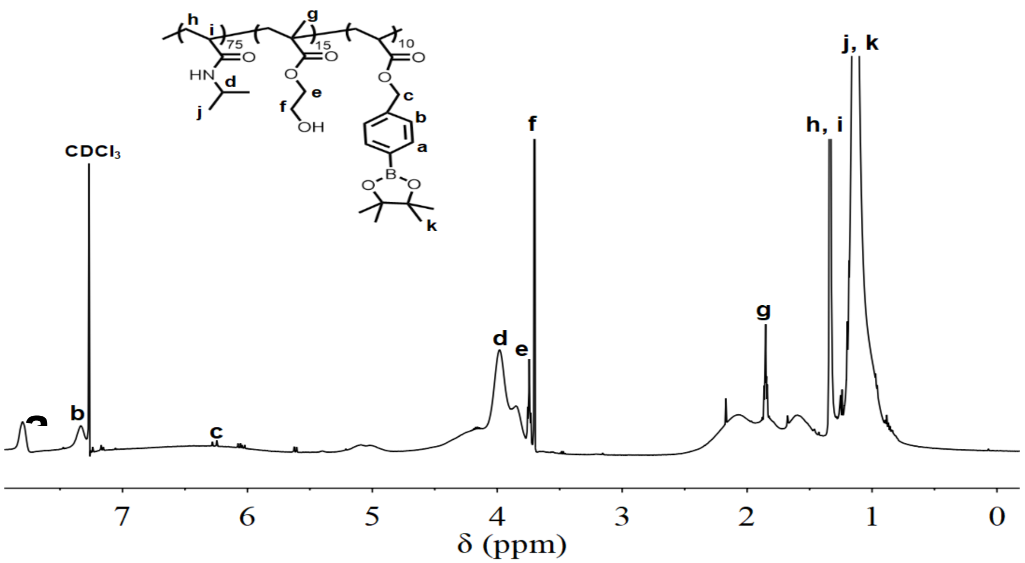

Supplementary Figure 6. ^{1}H-NMR spectrum of the hydrogel.

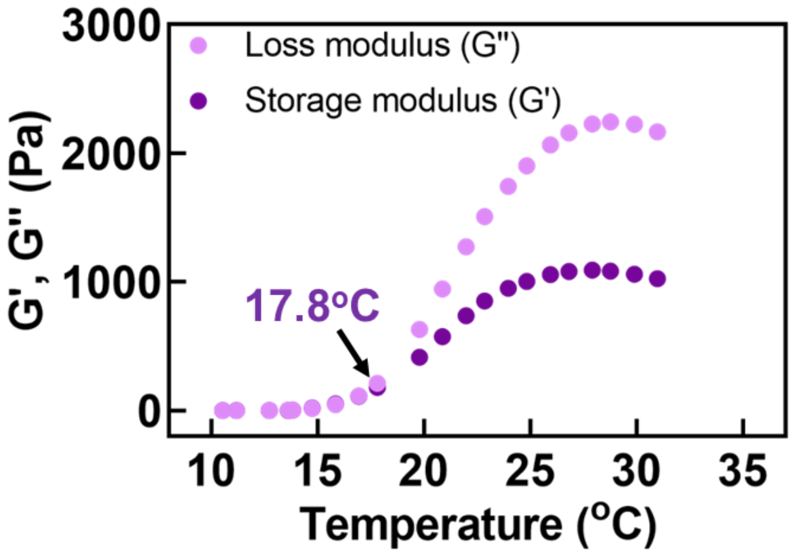

Supplementary Figure 7. Rheological test of the hydrogel at a fixed strain of 2% and frequency of 1 Hz using a Discovery HR-20 rheometer.

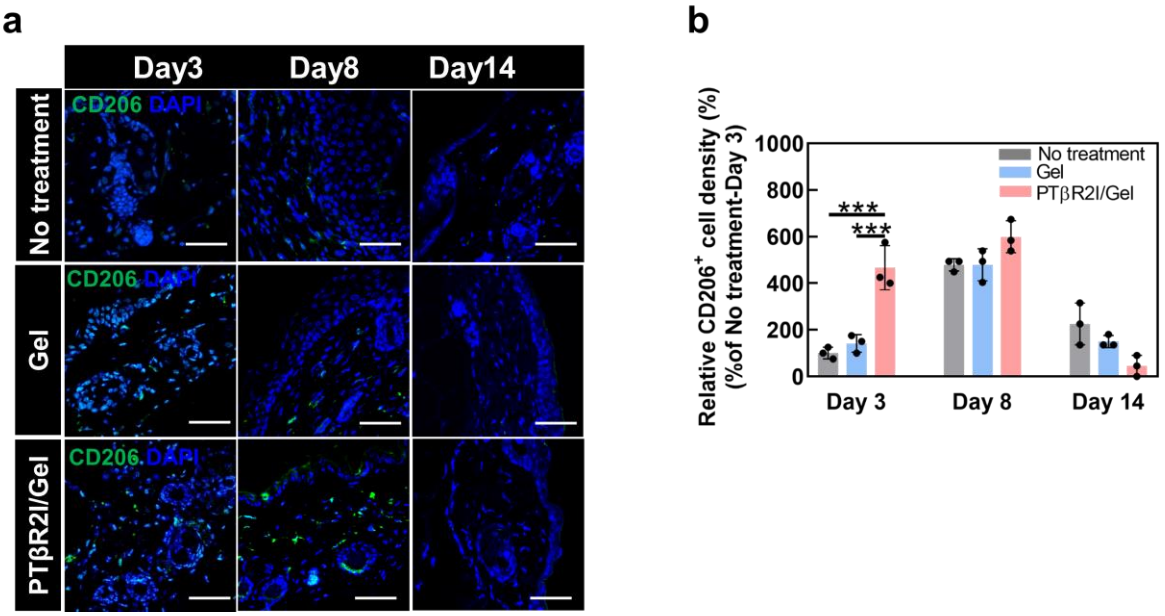

Supplementary Figure 8. Characterization of M2 macrophages in the wounds after 3, 8, and 14 days of treatment. **a.** Representative images of wounds stained for CD206 and DAPI. Scale bar = 50 μm. **b.** Quantification of relative $CD206^+$ cell density in the wounds (n=3).

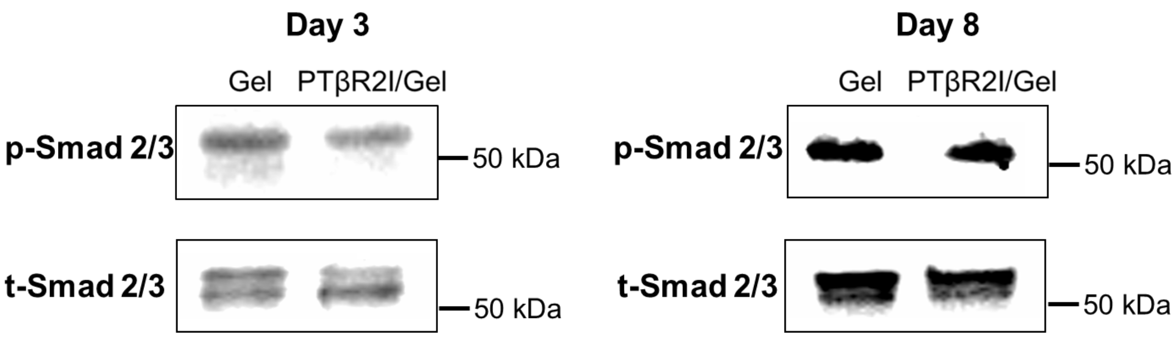

Supplementary Figure 9. Western blot analysis of p-Smad2/3 and t-Smad2/3 in the wounds of *db/db* mice at days 3 and 8.

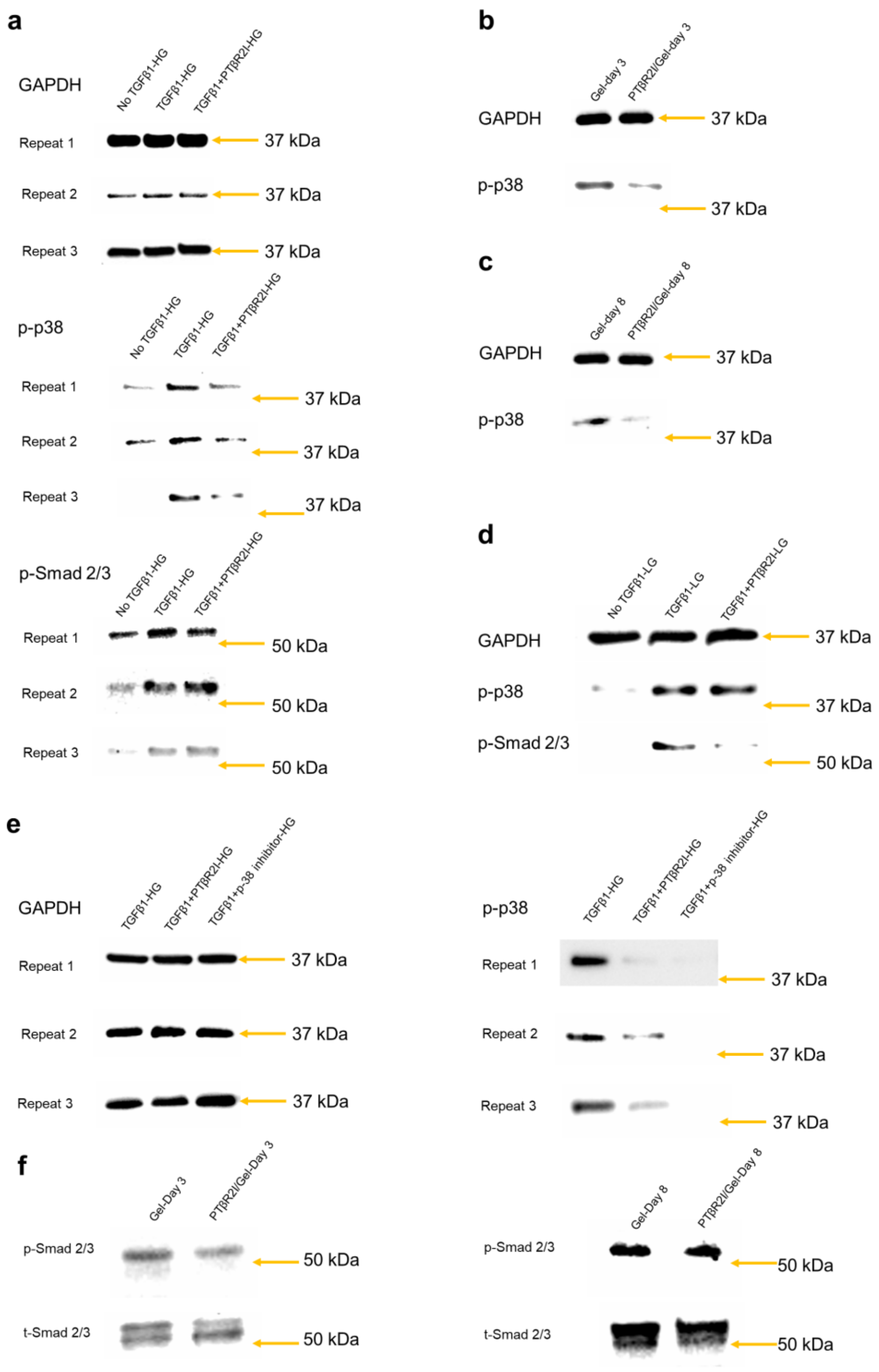

Supplementary Figure 10. Uncropped images of immunoblots in Figures. **a.** Images of blots for Figure 1v**.** **b.** Images of blot for Figure 5i. **c.** Images of blot for Figure 5j. **d.** Images of blot for Figure 7d. **e.** Image of blots for Supplementary Figure 3. **f.** Image of blots for Supplementary Figure 9.

Primer	Forward (5'-3')	Reverse (5'-3')	Species
IL1B	CTAAACAGATGAAGTGCTCC	GGTCATTCTCCTGGAAGG	Human
IL6	GCAGAAAAAGGCAAAGAATC	CTACATTTGCCGAAGAGC	Human
TNFA	ATGAGCACTGAAAGCATGATCC	GAGGGCTGATTAGAGAGAGG TC	Human
PDGFBB	GGGCAGGGTTATTTAATATGG	AATCAGGCATCGAGACAG	Human
VEGFA	AATGTGAATGCAGACCAAAG	GACTTATACCGGGATTTCTTG	Human
HGF	CAAGGACCTACGAGAAAATTAC	ATCACAGTTTGGAATTTGGG	Human
IGF1	CCCAGAAGGAAGTACATTTG	GTTTAACAGGTAACTCGTGC	Human

Supplementary Table 1. List of primer sequences used for real time RT-PCR.
