# Supplementary material for: Thermosensitive and antioxidant wound dressings capable of adaptively regulating TGFβ pathways promote diabetic wound healing
Source: NPJ Regen Med. 2023 Jul 8;8:32. doi: 10.1038/s41536-023-00313-3 (PMC10329719; doi:10.1038/s41536-023-00313-3)
Supplement: Supplementary file 2 — reporting summary [file 41536_2023_313_MOESM2_ESM.pdf]

nature portfolio

Corresponding author(s): Jianjun Guan

Last updated by author(s): Jun 21, 2023

Reporting Summary

Nature Portfolio wishes to improve the reproducibility of the work that we publish. This form provides structure for consistency and transparency in reporting. For further information on Nature Portfolio policies, see our Editorial Policies and the Editorial Policy Checklist.

Statistics

For all statistical analyses, confirm that the following items are present in the figure legend, table legend, main text, or Methods section.

n/a	Confirmed	
☐	☒	The exact sample size (*n*) for each experimental group/condition, given as a discrete number and unit of measurement
☐	☒	A statement on whether measurements were taken from distinct samples or whether the same sample was measured repeatedly
☐	☒	The statistical test(s) used AND whether they are one- or two-sided<br>*Only common tests should be described solely by name; describe more complex techniques in the Methods section.*
☒	☐	A description of all covariates tested
☒	☐	A description of any assumptions or corrections, such as tests of normality and adjustment for multiple comparisons
☐	☒	A full description of the statistical parameters including central tendency (e.g. means) or other basic estimates (e.g. regression coefficient) AND variation (e.g. standard deviation) or associated estimates of uncertainty (e.g. confidence intervals)
☐	☒	For null hypothesis testing, the test statistic (e.g. *F*, *t*, *r*) with confidence intervals, effect sizes, degrees of freedom and *P* value noted<br>*Give P values as exact values whenever suitable.*
☒	☐	For Bayesian analysis, information on the choice of priors and Markov chain Monte Carlo settings
☒	☐	For hierarchical and complex designs, identification of the appropriate level for tests and full reporting of outcomes
☒	☐	Estimates of effect sizes (e.g. Cohen's *d*, Pearson's *r*), indicating how they were calculated

Our web collection on statistics for biologists contains articles on many of the points above.

Software and code

Policy information about availability of computer code

Data collection	*Provide a description of all commercial, open source and custom code used to collect the data in this study, specifying the version used OR state that no software was used.*
Data analysis	*Provide a description of all commercial, open source and custom code used to analyse the data in this study, specifying the version used OR state that no software was used.*

For manuscripts utilizing custom algorithms or software that are central to the research but not yet described in published literature, software must be made available to editors and reviewers. We strongly encourage code deposition in a community repository (e.g. GitHub). See the Nature Portfolio guidelines for submitting code & software for further information.

Data

Policy information about availability of data

All manuscripts must include a data availability statement. This statement should provide the following information, where applicable:

- Accession codes, unique identifiers, or web links for publicly available datasets
- A description of any restrictions on data availability
- For clinical datasets or third party data, please ensure that the statement adheres to our policy

The data in the current study are available upon reasonable request.

Research involving human participants, their data, or biological material

Policy information about studies with human participants or human data. See also policy information about sex, gender (identity/presentation), and sexual orientation and race, ethnicity and racism.

Reporting on sex and gender	*Use the terms sex (biological attribute) and gender (shaped by social and cultural circumstances) carefully in order to avoid confusing both terms. Indicate if findings apply to only one sex or gender; describe whether sex and gender were considered in study design; whether sex and/or gender was determined based on self-reporting or assigned and methods used. Provide in the source data disaggregated sex and gender data, where this information has been collected, and if consent has been obtained for sharing of individual-level data; provide overall numbers in this Reporting Summary. Please state if this information has not been collected. Report sex- and gender-based analyses where performed, justify reasons for lack of sex- and gender-based analysis.*
Reporting on race, ethnicity, or other socially relevant groupings	*Please specify the socially constructed or socially relevant categorization variable(s) used in your manuscript and explain why they were used. Please note that such variables should not be used as proxies for other socially constructed/relevant variables (for example, race or ethnicity should not be used as a proxy for socioeconomic status). Provide clear definitions of the relevant terms used, how they were provided (by the participants/respondents, the researchers, or third parties), and the method(s) used to classify people into the different categories (e.g. self-report, census or administrative data, social media data, etc.) Please provide details about how you controlled for confounding variables in your analyses.*
Population characteristics	*Describe the covariate-relevant population characteristics of the human research participants (e.g. age, genotypic information, past and current diagnosis and treatment categories). If you filled out the behavioural & social sciences study design questions and have nothing to add here, write "See above."*
Recruitment	*Describe how participants were recruited. Outline any potential self-selection bias or other biases that may be present and how these are likely to impact results.*
Ethics oversight	*Identify the organization(s) that approved the study protocol.*

Note that full information on the approval of the study protocol must also be provided in the manuscript.

Field-specific reporting

Please select the one below that is the best fit for your research. If you are not sure, read the appropriate sections before making your selection.

☒ Life sciences ☐ Behavioural & social sciences ☐ Ecological, evolutionary & environmental sciences

For a reference copy of the document with all sections, see nature.com/documents/nr-reporting-summary-flat.pdf

Life sciences study design

All studies must disclose on these points even when the disclosure is negative.

Sample size	The sample size was not predetermined using statistical methods. For each experimental group, the number of animals/independent repeats was clearly indicated in the figures, and these repeats were deemed sufficient for conducting ANOVA analysis, with corresponding p-values calculated.
Data exclusions	No data was excluded from the analysis.
Replication	To ensure robustness of the results, all experiments were replicated using at least three independent tests. The number of specific repeats was also indicated in the corresponding figures.
Randomization	In each experiment, all samples were randomly assigned and analyzed together.
Blinding	Data analysis was performed by investigators who were blinded to the sample identities, and combined for each group afterwards.

Reporting for specific materials, systems and methods

We require information from authors about some types of materials, experimental systems and methods used in many studies. Here, indicate whether each material, system or method listed is relevant to your study. If you are not sure if a list item applies to your research, read the appropriate section before selecting a response.

Materials & experimental systems

n/a	Involved in the study	
☐	☒	Antibodies
☐	☒	Eukaryotic cell lines
☒	☐	Palaeontology and archaeology
☐	☒	Animals and other organisms
☒	☐	Clinical data
☒	☐	Dual use research of concern
☒	☐	Plants

Methods

n/a	Involved in the study	
☒	☐	ChIP-seq
☒	☐	Flow cytometry
☒	☐	MRI-based neuroimaging

Antibodies

Antibodies used

rabbit GAPDH antibody (Cell signaling, Cat# 2118), rabbit anti-α-smooth muscle actin (α-SMA, Cell signaling, Cat#19245), rabbit anti-phospho-Smad2(Ser465/467)/Smad3 (Ser423/425) (Cell signaling, Cat#8828), rabbit anti-phospho-p38 (Cell signaling, Cat# 4511), mouse anti-cytokeratin 14 (abcam, Cat#ab7800), rabbit anti-cytokeratin 10 (abcam, Cat#ab76318), rabbit anti-CD31 (abcam, Cat# ab28364), mouse anti-α-SMA (abcam, Cat# ab7817), rat anti-Ki67 (Thermofisher, Cat# MA5-14520), rabbit anti-CD86 (Cell Signaling, Cat# 91882), CellROX deep red (Thermofisher, Cat#C10422), Goat Anti-Mouse IgG H&L (HRP) (abcam, Cat# ab205719), Goat Anti-Rabbit IgG H&L (HRP) (abcam, Cat#ab205718), Alexa 647 goat anti-rabbit (Thermofisher, Cat# A-21245), Alexa 546 goat anti-mouse (Thermofisher, Cat# A-11003), and Alexa 488 goat anti-rabbit secondary antibodies (Thermofisher, Cat# A-11034).

Validation

The antibodies were acquired by consulting the manufacturer's website prior to purchase.
1. rabbit GAPDH antibody (Cell signaling, Cat# 2118)
https://www.cellsignal.com/products/primary-antibodies/gapdh-14c10-rabbit-mab/2118
2. rabbit anti-α-smooth muscle actin (α-SMA, Cell signaling, Cat#19245)
https://www.cellsignal.com/products/primary-antibodies/a-smooth-muscle-actin-d4k9n-xp-rabbit-mab/19245
3. rabbit anti-phospho-Smad2(Ser465/467)/Smad3 (Ser423/425) (Cell signaling, Cat#8828)
https://www.cellsignal.com/products/primary-antibodies/phospho-smad2-ser465-467-smad3-ser423-425-d27f4-rabbit-mab/8828
4. rabbit anti-phospho-p38 (Cell signaling, Cat# 4511)
https://www.cellsignal.com/products/primary-antibodies/phospho-p38-mapk-thr180-tyr182-d3f9-xp-rabbit-mab/4511
5. mouse anti-cytokeratin 14 (abcam, Cat#ab7800)
https://www.abcam.com/products/primary-antibodies/cytokeratin-14-antibody-ll002-ab7800.html
6. rabbit anti-cytokeratin 10 (abcam, Cat#ab76318)
https://www.abcam.com/products/primary-antibodies/cytokeratin-10-antibody-ep1607ihcy-cytoskeleton-marker-ab76318.html
7. rabbit anti-CD31 (abcam, Cat# ab28364)
https://www.abcam.com/products/primary-antibodies/cd31-antibody-ab28364.html
8. mouse anti-α-SMA (abcam, Cat# ab7817)
https://www.abcam.com/products/primary-antibodies/alpha-smooth-muscle-actin-antibody-1a4-ab7817.html
9. rat anti-Ki67 (Thermofisher, Cat# MA5-14520)
https://www.thermofisher.com/antibody/product/Ki-67-Antibody-clone-SP6-Recombinant-Monoclonal/MA5-14520
10. rabbit anti-CD86 (Cell Signaling, Cat# 91882)
https://www.cellsignal.com/products/primary-antibodies/cd86-e2g8p-rabbit-mab/91882
11. CellROX deep red (Thermofisher, Cat#C10422)
https://www.thermofisher.com/order/catalog/product/C10422?SID=srch-srp-C10422
12. Goat Anti-Mouse IgG H&L (HRP) (abcam, Cat# ab205719)
https://www.abcam.com/products/secondary-antibodies/goat-mouse-igg-hl-hrp-ab205719.html
13. Goat Anti-Rabbit IgG H&L (HRP) (abcam, Cat#ab205718)
https://www.abcam.com/products/secondary-antibodies/goat-rabbit-igg-hl-hrp-ab205718.html
14. Alexa 647 goat anti-rabbit (Thermofisher, Cat# A-21245)
https://www.thermofisher.com/antibody/product/Goat-anti-Rabbit-IgG-H-L-Highly-Cross-Adsorbed-Secondary-Antibody-Polyclonal/A-21245
15. Alexa 546 goat anti-mouse (Thermofisher, Cat# A-11003)
https://www.thermofisher.com/antibody/product/Goat-anti-Mouse-IgG-H-L-Cross-Adsorbed-Secondary-Antibody-Polyclonal/A-11003
16. Alexa 488 goat anti-rabbit secondary antibodies (Thermofisher, Cat# A-11034)
https://www.thermofisher.com/antibody/product/Goat-anti-Rabbit-IgG-H-L-Highly-Cross-Adsorbed-Secondary-Antibody-Polyclonal/A-11034
The selection of antibodies was based on their demonstrated reactivity to mouse and rat, as well as their suitability for use in immuno-fluorescence staining, histological staining, or immunoblotting. All of the antibodies employed in this study exhibited excellent performance.

Eukaryotic cell lines

Policy information about cell lines and Sex and Gender in Research

Cell line source(s)

HaCaT cells were purchased from AddexBio. Human dermal fibroblasts (HDFs) were purchased from Lonza. Human arterial endothelial cells (HAEC) were purchased from Cell Systems. THP-1 cells were purchased from ATCC.

Authentication

All cell lines were tested for authentication by their vendor.

Mycoplasma contamination	All cell lines tested negative for mycoplasma contamination.
Commonly misidentified lines (See ICLAC register)	This study did not involve misidentified cell lines.

Animals and other research organisms

Policy information about studies involving animals; ARRIVE guidelines recommended for reporting animal research, and Sex and Gender in Research

Laboratory animals	Female BKS.Cg-Dock7m+/+ Leprdb/J mice (db/db mice, Jackson Laboratories) and wild type (db/+) mice aged eight weeks were used for wound healing experiments. Female 8-week-old C57BL/6J mice were used for in vivo biocompatibility assay.
Wild animals	This study did not involve wild animals.
Reporting on sex	Female mice were used for the studies.
Field-collected samples	This study did not involve field-collected samples.
Ethics oversight	All animal study was performed under guidelines of the Washington University in St. Louis, Institutional Animal Care and Use Committee (IACUC), under approved protocol # 21-0186.

Note that full information on the approval of the study protocol must also be provided in the manuscript.
